# Effect of oxaliplatin plus 5-fluorouracil or capecitabine on circulating and imaging biomarkers in patients with metastatic colorectal cancer: a prospective biomarker study

**DOI:** 10.1186/s12885-021-08097-9

**Published:** 2021-04-01

**Authors:** Reem D. Mahmood, Danielle Shaw, Tine Descamps, Cong Zhou, Robert D. Morgan, Saifee Mullamitha, Mark Saunders, Nerissa Mescallado, Alison Backen, Karen Morris, Ross A. Little, Susan Cheung, Yvonne Watson, James P. B. O’Connor, Alan Jackson, Geoff J. M. Parker, Caroline Dive, Gordon C. Jayson

**Affiliations:** 1 Christie NHS Foundation Trust, Wilmslow Road, Withington, Manchester, M20 4BX UK; 2 The Clatterbridge Cancer Centre NHS Foundation Trust, Wirral, UK; 3 Cancer Research UK Manchester Institute Cancer Biomarker Centre, University of Manchester, Alderley Park, Macclesfield, UK; 4 Division of Cancer Sciences, School of Medicine, University of Manchester, Manchester, UK; 5 Division of Informatics, Imaging and Data Sciences, School of Health Sciences, University of Manchester, Manchester, UK; 6 Bioxydyn Limited, Manchester, UK; 7 Department of Computer Science, Centre for Medical Image Computing, University College London, London, UK

**Keywords:** Colorectal cancer, Angiogenesis, Biomarkers, Bevacizumab

## Abstract

**Background:**

Patients with metastatic colorectal cancer are treated with cytotoxic chemotherapy supplemented by molecularly targeted therapies. There is a critical need to define biomarkers that can optimise the use of these therapies to maximise efficacy and avoid unnecessary toxicity. However, it is important to first define the changes in potential biomarkers following cytotoxic chemotherapy alone. This study reports the impact of standard cytotoxic chemotherapy across a range of circulating and imaging biomarkers.

**Methods:**

A single-centre, prospective, biomarker-driven study. Eligible patients included those diagnosed with colorectal cancer with liver metastases that were planned to receive first line oxaliplatin plus 5-fluorouracil or capecitabine. Patients underwent paired blood sampling and magnetic resonance imaging (MRI), and biomarkers were associated with progression-free survival (PFS) and overall survival (OS).

**Results:**

Twenty patients were recruited to the study. Data showed that chemotherapy significantly reduced the number of circulating tumour cells as well as the circulating concentrations of Ang1, Ang2, VEGF-A, VEGF-C and VEGF-D from pre-treatment to cycle 2 day 2. The changes in circulating concentrations were not associated with PFS or OS. On average, the MRI perfusion/permeability parameter, *K*^trans^, increased in response to cytotoxic chemotherapy from pre-treatment to cycle 2 day 2 and this increase was associated with worse OS (HR 1.099, 95%CI 1.01–1.20, *p* = 0.025).

**Conclusions:**

In patients diagnosed with colorectal cancer with liver metastases, treatment with standard chemotherapy changes cell- and protein-based biomarkers, although these changes are not associated with survival outcomes. In contrast, the imaging biomarker, *K*^trans^, offers promise to direct molecularly targeted therapies such as anti-angiogenic agents.

**Supplementary Information:**

The online version contains supplementary material available at 10.1186/s12885-021-08097-9.

## Background

Colorectal cancer is the fourth most common cancer in the United Kingdom, with around 42,000 new cases diagnosed each year [1]. For patients presenting with metastatic disease, overall survival remains poor, with only around 10% alive 5 years after their diagnosis [2]. The management of patients with metastatic colorectal cancer has evolved over the past decade with the additional use of molecularly targeted therapies in combination with cytotoxic chemotherapy.

For patients diagnosed with colorectal cancer with liver metastases, first line standard cytotoxic chemotherapy includes: FOLFOX (5-fluorouracil/folinic acid plus oxaliplatin), FOLFIRI (5-fluorouracil/folinic acid plus irinotecan) or CAPOX (capecitabine plus oxaliplatin) [3, 4]. Targeted therapies against vascular endothelial growth factors (VEGF) e.g. bevacizumab, and epidermal growth factor receptors (EGFR) e.g. cetuximab or panitumumab, are also recommended for first line management of patients with metastatic colorectal cancer in combination with cytotoxic chemotherapy [3, 4]. Indeed, bevacizumab, cetuximab and panitumumab have been shown in randomised phase III trials to prolong progression-free survival (PFS) [5–13] and overall survival (OS) [5–7, 12]. Circulating biomarkers, such as CK18, have been shown to be predictive of prognosis and progression in colorectal cancer [14, 15]. In addition, use of genetics can guide the use of EGFR treatment, with patients that are KRAS/BRAF mutant not benefitting from these drugs [16–18]. However, there are no validated circulating or imaging biomarkers to guide the use of VEGF and EGFR therapies, which are expensive and associated with toxicity.

Early phase trials have assessed the effect of traditional cytotoxic chemotherapy in combination with bevacizumab using magnetic resonance imaging (MRI) [19–22] and circulating biomarkers [23–25]. However, in order to better understand the data reported for combination therapy, the effects of cytotoxic chemotherapy alone need to be assessed as a control. In this study, MRI and blood-based biomarkers were investigated in patients undergoing standard cytotoxic chemotherapy. Data from this study may improve the understanding of the utility of these biomarkers for future trials incorporating molecularly targeted therapies.

## Methods

This was a prospective, single-centre, biomarker-driven study recruiting patients that were treated at the Christie NHS Foundation Trust for colorectal cancer with liver metastases. Ethical approval was obtained from the local ethics committee (see Supplementary Information). All patients gave written informed consent to participate in the study.

### Study participants

Eligible participants included those with histologically-proven colorectal cancer; liver metastases measuring at least 30 mm in the longest axis; 18 years of age or older; a World Health Organization (WHO) performance status of 0 to 2; were planned to commence primary therapy with oxaliplatin plus 5-fluorouracil (5-FU) or capecitabine; white cell blood count ≥4 × 10^9^/l; platelet count ≥100 × 10^9^/l; serum total bilirubin concentration ≤ 1.5 × upper limit of normal (ULN); serum alkaline phosphatase concentration ≤ 5 × ULN and; a calculated glomerular filtration rate ≥ 50 ml per minute.

Patients were excluded if MRI was contra-indicated due to standard criteria relating to metal implants or allergy to MRI contrast; use of adjuvant chemotherapy within 12 months prior to study enrolment; a personal medical history including any non-colorectal malignancy within 5 years of study enrolment; concurrent use of other investigational medicinal product or; pregnant or breast-feeding women.

### Study drugs

Patients were treated with either oxaliplatin plus 5-FU (oxaliplatin 85 mg/m^2^ of body surface area [BSA] plus folinic acid 350 mg and 5-FU 400 mg/m^2^ on day 1 followed by 5-FU 2400 mg/m^2^ intravenous infusion [46 h] every two-week cycle) or oxaliplatin plus capecitabine (oxaliplatin 130 mg/m^2^ on day 1 and capecitabine 1000 mg/m^2^ on day 1 to 14 every 3-week cycle) for a maximum of 6 cycles.

### Clinical endpoints

Clinical endpoints included progression-free survival (PFS) and overall survival (OS). Progressive disease was defined as the time interval from the date of study registration to the date of either clinical or radiological progression or death. On imaging, progressive disease was measured using the response evaluation criteria in solid tumours (RECIST) version 1.1 [26]. OS was defined as the time interval from the date of study registration to the date of death. All patients were followed up until they reached the PFS efficacy endpoint; no censoring was present in the dataset.

Computed tomography (CT) was performed every 8 weeks as part of standard tumour assessment. As part of standard treatment, plasma carcinoembryonic antigen (CEA) and lactate dehydrogenase (LDH) concentrations were measured at the start of each cycle of chemotherapy. Both can be used to predict prognosis and response to treatment in metastatic colorectal cancer [27, 28].

### Biomarker schedule

A detailed description of the methodology used for the imaging and circulating biomarkers is provided in the Supplementary information.

Study time points for dynamic contrast-enhanced MRI (DCE-MRI) and diffusion weighted MRI (DW-MRI) included pre-treatment, cycle 1 day 2, cycle 1 day 8, cycle 2 day 2 of chemotherapy and following 12 weeks of chemotherapy. At pre-treatment, MRI scans were carried out twice, at least 24 h apart, to determine the repeatability of the imaging biomarkers. Regions of interest (ROIs) within the liver were defined manually by a trained operator, in order to determine whole tumour volume (WTV) from T_1_- and T_2_-weighted images as well as the DCE-MRI images. Parameters derived from DCE-MRI included the transfer coefficient (*K*^trans^), volume of extravascular extracellular space (ν_e_) and vascular plasma volume (ν_p_). For DWI-MRI, the apparent diffusion coefficient (ADC) was derived.

Blood samples for circulating tumour cells (CTCs) and a panel of plasma-derived circulating protein biomarkers were collected at the same time points as MRI including Ang2, VEGF-A, VEGF-C, VEGF-D, VEGFR1, VEGFR2, IL6, IL8, Tie2, KGF, PlGF, FGFb, HGF, PDGFbb, SDF1b, E-selectin, M65 and VCAM-1.

### Statistical analysis

The target recruitment for the study was 20 patients. All biomarkers were assessed for normality and transformed when necessary. To identify whether biomarker concentrations changed significantly from pre-treatment to cycle 2 day 2, paired Student’s t-tests were performed. Cycle 2 day 2 of chemotherapy was selected for significance testing in order to determine the early effects of cytotoxic chemotherapy. A correlation network analysis was performed to examine the relationship between multiple biomarkers without the requirement to conduct multiple sequential analyses [23, 25]. This was done based on Pearson correlations and build from the qgraph package in R.

Cox proportional hazard regression was used for survival analysis, respecting the proportionality and linearity assumptions. Kaplan Meier curves were constructed using dichotomized data (longitudinal increase versus decrease in biomarker concentration), and the median PFS and OS intervals in each group were calculated. Statistical significance was determined using *p*-values, with a cut off of 0.025 being considered statistically significant in order to reduce the impact of multiple testing. More stringent adjustment for multiple comparisons was not considered due to the limited sample size. Analysis was carried out using R 3.5.0.

## Results

### Patient characteristics

Between October 2011 and November 2013, 20 patients were recruited to the study. Patient demographics are shown in Table 1. The mean age of participants was 69 years and the majority were male (85%). During the study, the best radiological response to chemotherapy included: 12 patients (60%) had RECIST complete or partial response (CR/PR), 2 patients (10%) had RECIST stable disease (SD) and 6 patients (30%) had disease progression. Across the entire cohort, the median PFS and OS were 8.7 and 17.3 months, respectively. Twelve patients completed all scanning protocols at chemotherapy cycle 6 and attrition occurred due to falling performance status throughout the trial. The imaging protocols were well tolerated and provided repeatable results.

**Table 1:** Pre-treatment patient demographics

Patient demographic	Value
Total patients	20
Sex: number (percentage)	
Male	17 (85%)
Female	3 (15%)
Age: (years)	
Mean	69
Range	58–80
WHO performance status: number (percentage)	
0	7 (35%)
1	12 (60%)
2	1 (5%)
Pre-treatment CEA (μg/L):	
Mean	384
Range	3–2897
Pre-treatment LDH (IU/L):	
Mean	2279
Range	45–11,346
Chemotherapy regimen: number (percentage)	
Oxaliplatin and 5FU	18 (90%)
Oxaliplatin and capecitabine	2 (10%)

### Pre-treatment biomarkers

Pre-treatment characteristics including age, WHO performance status and pre-treatment concentrations of CEA and LDH were not associated with PFS or OS. Pre-treatment, CTCs were detectable in all 20 patients. The mean number of CTCs was 4 per 7.5 ml of blood.

Evaluation of the association between survival outcomes and pre-treatment concentrations of circulating biomarkers showed that lower concentrations of Ang2 (HR 0.41, 95%CI 0.19–0.86, *p* = 0.019) and VEGF-A (HR 0.41, 95%CI 0.19–0.87, *p* = 0.021) were associated with a significantly reduced PFS. No other pre-treatment biomarkers were found to be associated with PFS or OS.

### Biomarkers on treatment

Data showed that the plasma concentration of most circulating angiogenesis-related biomarkers reduced from pre-treatment to cycle 2 day 2, with significant reductions in Ang1, Ang2, VEGF-A, VEGF-C, and VEGF-D (Table 2). VCAM-1 was the only circulating biomarker to significantly increase (*p* = 0.0194). The increase of VCAM-1 and decrease of all other circulating biomarkers from pre-treatment to cycle 2 day 2 was not associated with PFS or OS.

**Table 2:** Significant changes in circulating and imaging biomarkers from pre-treatment to cycle 2 day 2

Biomarker type	Biomarker name	Mean difference from pre-treatment to C2D2 [95% CI]	***p***-value
Circulating	VEGF-C	−0.932 [−1.333, −0.531]	0.0002
	FGFb	−0.866 [− 1.262, − 0.469]	0.0003
	VEGF-A	−0.788 [− 1.16, − 0.415]	0.0004
	M65	− 0.57 [− 0.845, − 0.296]	0.0004
	Ang2	− 0.722 [− 1.084, − 0.36]	0.0006
	Ang1	− 0.723 [− 1.101, − 0.345]	0.0009
	VEGF-D	− 0.316 [− 0.489, − 0.143]	0.0014
	CTCs	− 1.313 [−2.074, − 0.552]	0.0021
	PDGFbb	−0.44 [− 0.74 - -0.14]	0.0070
	IL8	−0.47 [− 0.82 - -0.12]	0.0124
	VCAM-1	0.32 [0.07–0.58]	0.0149
	E-selectin	−0.3 [− 0.54 - -0.06]	0.0183
Imaging	WTV (mm^3^)	−0.523 [− 0.751, − 0.295]	0.0002
	ETV (mm^3^)	−0.56 [− 0.813, − 0.307]	0.0003
	T1 (ms)	− 0.144 [− 0.217, − 0.071]	0.0008
	ADC (×10^−3^mm2/s)	0.01 [0.00–0.02]	0.0170
	ν_e_	0.04 [0.01–0.07]	0.0254
	*K*^trans^ (/min)	3.38 [0.05–6.70]	0.0469 *

Biomarker measurements were all log2 transformed, except ADC and *K*^trans^ which were multiplied by 100, and iAUC, ν_e_, ν_p_ and EF which were not changed**.** Analysed using paired Student’s t-test to assess for statistical significance

All circulating biomarkers measured in pg/ml. Imaging biomarkers are stated. ν_e_ has no units

*Not statistically significant based on study cut-off of *p* value < 0.025

The mean number of CTCs significantly reduced from pre-treatment to cycle 2 day 2 (*p* = 0.0021). A higher number of CTCs at cycle 2 day 2 was associated with significantly worse OS (HR 2.82, 95%CI 1.3–6.1, *p* = 0.008).

**Fig. 1:**
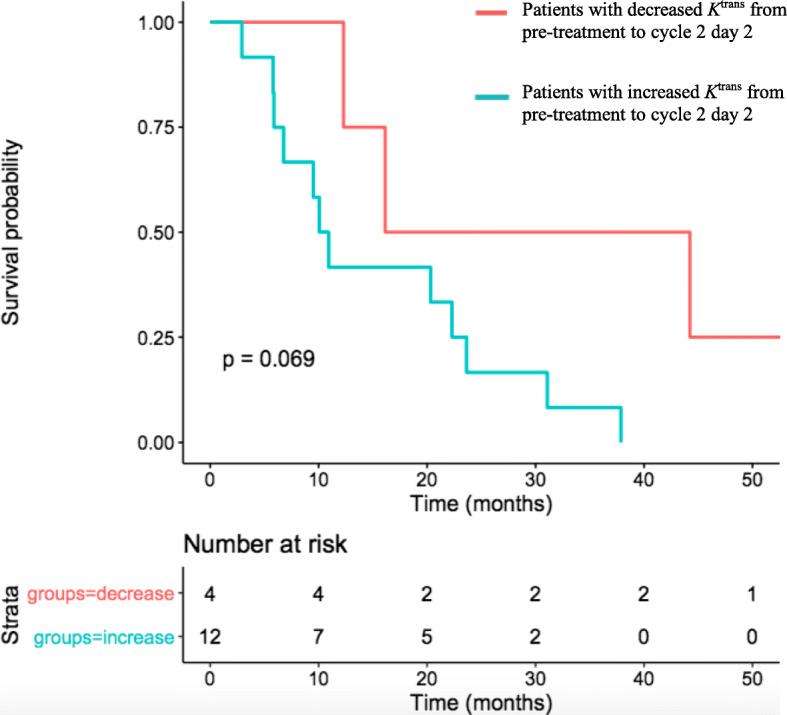
Kaplan Meier Curve to show overall survival for patients with an increase in *K*trans from pre-treatment to cycle 2 day 2 compared with patients with a decrease in *K*trans from pretreatment to cycle 2 day 2

MRI data showed that WTV, enhancing tumour volume (ETV) and T1 decreased significantly from pre-treatment to cycle 2 day 2 (p = 0.002, *p* = 0003 and *p* = 0008, respectively). The ADC significantly increased from pre-treatment to cycle 2 day 2 (*p* = 0.017) (Table 2). However, none of these parameters were associated with PFS or OS. On average, there was an increase in *K*^trans^ from pre-treatment to cycle 2 day 2. In those patients whose *K*^trans^ increased at cycle 2 day 2, there was a significantly worse OS outcome when compared to those patients whose *K*^trans^ did not increase at cycle 2 day 2 (HR 1.099, 95%CI 1.01–1.20, *p* = 0.025) (Fig. 1).

All patients had an increased CEA concentration from pre-treatment to cycle 2, but this was not associated with PFS nor OS (PFS: *p* = 0.521, OS: *p* = 0.638). There was no significant difference between the mean increase in CEA concentration between patients with an increased *K*^trans^ and those with a decreased *K*^trans^ (6.79 [95% CI 6.17–7.36] versus 6.51 [95% CI 6.21–6.81], respectively, *p* = 0.43).

**Fig. 2:**
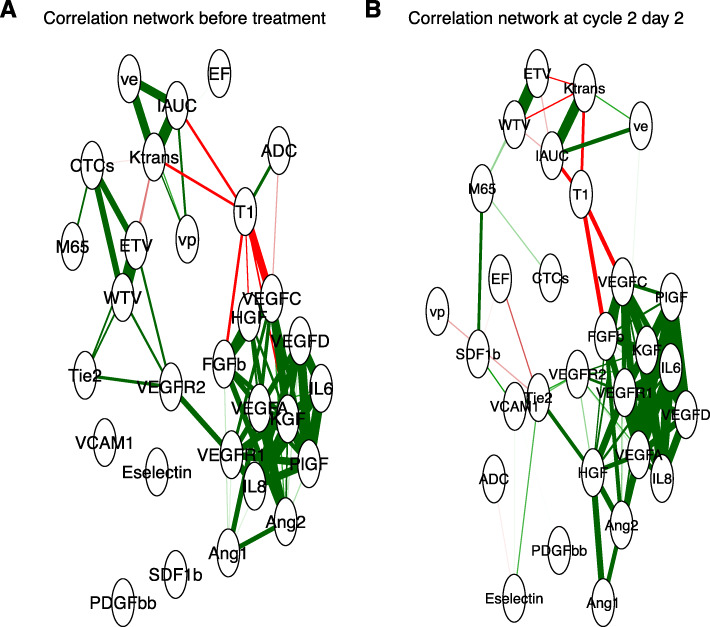
Correlation network to compare correlation between biomarkers at pre-treatment and at cycle 2 day 2. Correlation networks show the positive (green) and negative (red) correlations between biomarkers. The plot shows that both at pre-treatment and at cycle 2 day 2 the angiogenic biomarkers cluster closely together in a network

The correlation network analysis showed that, across all patients, the change in the circulating concentrations of angiogenesis-related proteins in response to chemotherapy was similar. The interaction between these proteins is undisturbed by cytotoxic chemotherapy, shown by the close clustering of angiogenic biomarkers both at pre-treatment and at cycle 2 day 2 (Fig. 2).

See Supplementary Information for the full data set.

## Discussion

In this study we have investigated the impact of cytotoxic chemotherapy on circulating proteins and CTCs as surrogate markers of tumour vasculature and cellularity, in parallel with MRI, to document changes in tumour perfusion and tumour volume. This study was limited by the small numbers of participants and so should be regarded as exploratory only.

A key finding of this study was that an increase in *K*^trans^ at cycle 2 day 2 was associated with a worse OS. In contrast, trials of anti-angiogenic or vascular disrupting agents show near universal early decrease in *K*^trans^ [19]. This is considered to reflect reduction in either perfusion or permeability of tumour blood vessels, or a combination of the two processes, rather than a systemic effect. The process by which *K*^trans^ changes with a cytotoxic chemotherapy regimen is more complex and indirect. It is possible that is that in these patients, the tumour adapts to chemotherapy by increasing in its vasculature. Another possibility is that these patients have tumours which partly respond to chemotherapy, as shown by the reduction in WTV, but the remaining tumour mass consists of well-perfused chemotherapy-resistant tissue. This process could be explained by a “healing response” to chemotherapy, causing an increase in tumour vascular function and reflected by an increased *K*^trans^ value. As the network analysis does not describe changes that are associated with individual biomarkers in isolation, but rather global reductions in angiogenesis biomarkers, the implication is that there is not a dynamic biological response to chemotherapy. Thus, chemotherapy is killing sensitive tumour cell populations leaving behind more resistant clones; characterised here with high *K*^trans^. However, since massive cell death of both tumour and non-tumour cells resulting from chemotherapy can lead to the release of damage associated molecular pattern (DAMPs) proteins, it is possible that any resultant systemic inflammatory response could lead to increase in vasculature permeability, detected an increased *K*^trans^.

Interestingly, the standard of care marker of treatment response, CEA concentration, increased in all patients from pre-treatment to cycle 2. As mentioned, there was no significant difference between the mean increase in CEA concentration between patients with an increased *K*^trans^ and those with a decreased *K*^trans^. This suggests that *K*^trans^ could be more useful than CEA to predict poorer responses to cytotoxic chemotherapy. This finding may also direct earlier introduction of anti-angiogenic agents, such as bevacizumab.

This study also shows an association between lower pre-treatment circulating concentrations of Ang2 and VEGF-A and worse PFS. There is inconsistency in the value of pre-treatment biomarkers of angiogenesis reported in the literature [29–31]. Results differ between studies (Table 3) but are also difficult to compare due to different angiogenesis-related proteins investigated and methods of sampling/analysis used. Hence, our study focussed on the significance of dynamic response to treatment rather than pre-treatment concentrations.

**Table 3:** Correlation between pre-treatment circulating angiogenesis-related biomarkers and survival outcomes in studies which recruited patients with metastatic colorectal cancer [22–24]

Study Title	No. of patients	Treatment received	Pre-treatment biomarker	Correlation with survival outcomes
Prognostic/predictive value of 207 serum factors in colorectal cancer treated with cediranib and/or chemotherapy^a^ [22]	582	FOLFOX or CAPOX chemotherapy + cediranib/placebo	VEGF-D	Low pre-treatment concentration correlated with improved PFS and OS regardless of treatment received
			VEGFR-1	
			VEGFR-3	
			Tie-2	
			Ang2	No correlation
Changes in circulating VEGF levels in relation to clinical response during chemotherapy for metastatic cancer [23]	90	Camptothecin	VEGF-165	Patients with high pre-treatment concentration were more likely to have progressive disease during treatment
Phase II Trial of Infusional Fluorouracil, Irinotecan, and Bevacizumab for Metastatic Colorectal Cancer: Efficacy and Circulating Angiogenic Biomarkers Associated With Therapeutic Resistance [24]	43	FOLFIRI + bevacizumab	VEGF-2	No correlation

^a^As part of the phase III, Horizon II trial

## Conclusion

In conclusion, the response to cytotoxic chemotherapy treatment in patients with colorectal cancer with liver metastases showed a maintained robust relationship between angiogenic biomarkers. In some patients, poor outcome was associated with the early detection of well-perfused tissue in smaller tumours suggesting that chemotherapy was unable to kill the remaining component of a tumour, presumably because of increased clearance of cytotoxic agents. These findings identify a group of patients whose tumour does not respond well to traditional cytotoxic chemotherapy alone and who might benefit from early addition of molecularly targeted therapies.

## Supplementary Information

**Additional file 1.**

## Data Availability

The datasets used and/or analysed during the current study are available from the corresponding author on reasonable request.

## Abbreviations

5-FU: Fluorouracil
ADC: Apparent diffusion coefficient
Ang: Angiopoetin
BSA: Body surface area
CAPOX: Capecitabine plus oxaliplatin
CEA: Carcinoembryonic antigen
CI: Confidence interval
CK18: Cytokeratin-18
CT: Computed tomography
CTC: Circulating tumour cells
CR: Complete response
DAMPs: Damage associated molecular pattern
DCE-MRI: Dynamic contrast-enhanced magnetic resonance imaging
DWI-MRI: Diffusion weighted magnetic resonance imaging
EGFR: Epidermal growth factor receptors
ETV: Enhancing tumour volume
FGFb: Basic fibroblastic growth factor
FOLFIRI: Fluorouracil/folinic acid plus irinotecan
FOLFOX: Fluorouracil/folinic acid plus oxaliplatin
HGF: Hepatocyte growth factor
HR: Hazard ratio
iAUC: Incremental area under the curve
IL8: Interleukin 8
KGF: Keratinocyte growth factor
*K*^trans^: Endothelial contrast agent transfer coefficient
LDH: Lactate dehydrogenase
MRI: Magnetic resonance imaging
NHS: National Health Service
OS: Overall survival
PDGFbb: Platelet derived growth factor beta
PFS: Progression-free survival
PlGF: Placental growth factor
PR: Partial response
RECIST: Response evaluation criteria in solid tumours
ROI: Regions of interest
SD: Stable disease
SDF1b: Stromal cell-derived factor 1
ULN: Upper limit of normal
VCAM1: Vascular cell adhesion molecule 1
ν_e_: Fractional extravascular extracellular volume
VEGF: Vascular endothelial growth factor
VEGFR: Vascular endothelial growth factor receptor
ν_p_: Fractional blood plasma volume
WHO: World Health Organization
WTV: Whole tumour volume
