# Supplementary material for: Effect of oxaliplatin plus 5-fluorouracil or capecitabine on circulating and imaging biomarkers in patients with metastatic colorectal cancer: a prospective biomarker study
Source: BMC Cancer. 2021 Apr 1;21:354. doi: 10.1186/s12885-021-08097-9 (PMC8017714; doi:10.1186/s12885-021-08097-9)
Supplement: Supplementary file 1 — Additional file 1. [file 12885_2021_8097_MOESM1_ESM.doc]

**SUPPLEMENTARY INFORMATION**

**Contents**

1. Methods
2. Results
3. References
4. **METHODS**

**Ethical approval**

This trial was performed with local ethics committee approval in accordance with the UK Clinical Trials regulations for compliance with Good Clinical Practice Guidelines (NHS Research Ethics Service Committee North West - Liverpool East, 11/NW/0118)(1). Local research and development department approval was obtained and laboratory work was carried out in accordance with the principles of Good Clinical Laboratory Practice (World Health Organization 2009).

**MRI Acquisition**

Pre-treatment MRI scans were performed twice before commencing chemotherapy, a minimum of 24 hours apart, to assess the reproducibility of measurements and thus the impact of treatment on imaging parameters. The same protocol was then repeated on cycle 1 day 2; cycle 1 day 8, cycle 2 day 2 and after 12 weeks of treatment, where cycle 1 day 1 was defined as the day of the first chemotherapy dose. DW-MRI and DCE-MRI were performed in the same scanning session.

Imaging was performed using a 1.5 T Philips Achieva scanner (*Philips Healthcare, Best, The Netherlands*) at the Wolfson Molecular Imaging Centre, University of Manchester. DCE-MRI was carried out as previously described (2).

The field of view (FOV) was centred on the liver. In each examination, *T*_1_-weighted fast field echo images (TR=10 ms, TE=4.6 ms, *α*=15°) and *T*_2_-weighted single shot turbo spin echo images (TR=606.5 ms, TE=80 ms, *α*=90°) were acquired. Both sequences employed field of view (FOV) 375×375 mm^2^, matrix 256×256 with a 4-mm slice thickness.

For the DCE-MRI series, 75 3-dimensional axial volumes were acquired consecutively (TR=4.0 ms, TE=0.82 ms, *α*=20°, one signal average, FOV of 375×375 mm^2^, matrix 128×128; in-plane voxel size 2.93×2.93 mm^2^) following calculation of baseline *T*_1_ using the variable flip angle method (Fram et al 1987) (*α*=2°/10°/20°; four signal averages; identical TR, TE, imaging matrix, and slice thickness). Temporal resolution was 4.97 s during the dynamic sequence, 0.1 mmol/kg of gadoterate meglumine contrast agent (*Dotarem, Guebert, France*) was administered intravenously at the 6^th^ dynamic time-point at a rate of 3 ml/second, using a Medrad Spectris power injector (*Bayer Healthcare Pharmaceuticals, USA*).

DWI-MR images were acquired using a non-breath holding, fat-suppressed pulsed gradient spin-echo (PGSE) echo-planar imaging sequence with the following parameters: FOV 375 mm × 375 mm; slice thickness 4 mm; number of slices 25; matrix 256 × 256; in-plane resolution 1.46 mm × 1.46 mm; TR 3416 ms; TE 90 ms with *b* values of 0, 150, 500 and 800 s/mm².

**MRI Analysis**

Regions of interest (ROIs) were defined manually by an experienced operator for the WTV using Java Image software (*JIM version 5.0, Xinpase Systems Ltd, UK*) and with reference to the T_1_- and T_2_-weighted images as well as the DCE-MRI images. The arterial input function (AIF) was determined for each patient visit using an automated technique in the nearest feeding artery (3). An assumed haematocrit of 0.42 was input into the modelling process. Output parameters included whole tumour volume (WTV), tumour enhancing volume, tumour enhancing fraction, and tumour median values of the initial area under the DCE-MRI contrast agent concentration time course at 60 s (iAUC_60_), the endothelial contrast agent transfer coefficient (*K*^trans^)*,* fractional blood plasma volume (*v*_p_), and the fractional extravascular extracellular volume (*v*_e_), as derived using the extended Kety model (4).

All DCE-MRI analyses were performed using in-house software, Manchester Dynamic Modelling (MaDyM). DW-MRI was analysed by fitting the tumour ROI data to obtain the water apparent diffusion coefficient (ADC). Voxel-wise estimates of ADC were calculated by fitting the image data to S(b) = S_0_e^-b*ADC^, where *b* is the *b* value for each DWI acquisition, using in-house written software. ADC maps were generated and median tumour ADC was calculated to summarise each tumour.

**Circulating Tumour Cells**

Whole blood was drawn for enumeration of CTCs at time-points corresponding to the MRI scanning schedule. The aim was to test the hypothesis that vascular changes seen on DCE-MRI correlate with the number of tumour cells released into the circulation. Ten millilitres of blood were drawn into a CellSave preservation tube and CTC analysis was carried out within 96 hours, using the FDA-approved CellSearch technology tube (*Janssen Diagnostics, UK*). The CellSearch CellTracks AutoPrep System (*Janssen Diagnostics, UK*) was used to prepare the samples and the Cell Search CellTracks Analyzer II CellTracks (*Janssen Diagnostics, UK*) used to image the cells. The CellSearch CTC Kit (*Janssen Diagnostics, UK*) contained ferrofluid particles coated with antibodies to epithelial cell adhesion molecule (EpCAM) to separate cells of epithelial origin immunomagnetically from blood. Leucocytes were excluded by using a fluorescent antibody to CD45. Epithelial cells were identified through antibodies to cytokeratins 8, 18 and 19 and a nuclear dye, 4, 6-diamidino-2-phenylindole (DAPI) (5). Cells were classified as CTCs if they were CD45 negative and positive for DAPI staining and cytokeratins. Sample processing and analysis were carried out strictly according to the manufacturer’s instructions.

**Circulating angiogenesis-associated proteins**

Blood was drawn for quantification of circulating angiogenesis-associated proteins at time-points corresponding to the MRI scans to test the hypothesis that vascular changes seen on DCE-MRI would correlate with the concentration of circulating angiogenic factors. Blood samples for measuring angiogenic markers were drawn directly into 6ml EDTA vacutainers and plasma was separated from the blood cells by centrifugation of the sample at 3000*g* for 10 minutes. Enzyme-Linked Immuno-Sorbent Assays (ELISAs) were performed using SearchLight chemiluminescent arrays and SearchLight Plus charged couple device imaging system (*Aushon Biosystems [now Quanterix Corp], Boston, US*). These are multiplex assays that can be used for the quantification of up to six different proteins in each well of a ninety-six well plate. This multiplex approach allowed a greater number of analytes to be investigated using a small blood volume. All assays were performed at the Clinical and Experimental Pharmacology GCPL laboratories, Cancer Research UK: Manchester Institute and were subject to in-house validation as previously described (6). Protein concentrations were assessed using six-plex ELISAs of Ang2, FGFb, HGF, PDGFbb, VEGF-A and VEGF-C and IL6, IL8, KGF, PlGF, VEGFR1, VEGFR2; duplexes of Ang1 and Tie2 and E-selectin and VCAM-1 and single plexes of VEGFD and SDF1b.

**Circulating markers of cell death**

CK18 is a member of the intermediate filament family of cytoskeletal proteins and is widely expressed in epithelial and endothelial cells. The M65 ELISA measures total soluble CK18 which is released during apoptotic and necrotic forms of cell death. M65 has been validated as a marker of cell death in colorectal cancer (7) and was examined in this study alongside the diffusion weighted imaging data, to explore the relationship between circulating and imaging biomarkers of cell death. Blood was collected in tubes containing a silica clot activator and centrifuged at 2000*g* for 10min to obtain serum for analysis. The M65 ELISA assay (*Peviva Stockholm*) is a commercially available ELISA kit and experiments were carried out according to the manufacturer’s instructions, as described previously (8). Plasma carcinoembryonic antigen (CEA) and lactate dehydrogenase (LDH) were also measured at these time-points as part of routine hospital standard of care blood testing.

1. **RESULTS**

**Pre-treatment patient demographics**

***Table 1: Pre-treatment patient demographics associated with overall survival***

No pre-treatment patient demographics were significantly associated with overall survival.

*CEA and LDH were log2 transformed

| **Patient Demographic** | **HR [95% CI]** | **p-value** |
| --- | --- | --- |
| LDH (IU/L) * | 1.2 [0.87 - 1.65] | 0.26 |
| Histology | 1.57 [0.5 - 4.96] | 0.42 |
| CEA (µg/L) * | 0.94 [0.76 - 1.15] | 0.52 |
| Age | 1.03 [0.95 - 1.11] | 0.53 |
| WHO performance status | 0.78 [0.29 - 2.14] | 0.63 |

***Table 2: Pre-treatment patient demographics associated with progression free survival***

No pre-treatment patient demographics were significantly associated with progression free survival.

*CEA and LDH were log2 transformed

| **Patient Demographic** | **HR [95% CI]** | **p-value** |
| --- | --- | --- |
| Histology | 0.4 [0.12 - 1.4] | 0.15 |
| WHO performance status | 0.51 [0.18 - 1.45] | 0.2 |
| CEA (µg/L) * | 1.02 [0.86 - 1.21] | 0.83 |
| LDH (IU/L) * | 0.97 [0.71 - 1.33] | 0.86 |
| Age | 1 [0.92 - 1.09] | 0.99 |

**Pre-treatment biomarkers**

***Table 3: Pre-treatment biomarker measurements***

Biomarker measurements were all log2 transformed, except ADC and *K*^trans^ which were multiplied by 100, and iAUC, ν_e_, ν_p_ and EF which were not changed.

All circulating biomarkers measured in pg/ml. Imaging biomarkers are stated. ν_e_, ν_p_ and EF have no units.

| **Biomarker type** | **Biomarker name** | **Actual median** | **Actual range** | **Transformed median*** | **Transformed range*** |
| --- | --- | --- | --- | --- | --- |
| Circulating | IL6 | 22.21 | 5.54 - 412.4 | 4.51 | 2.69 - 8.69 |
|  | IL8 | 70.76 | 16.56 - 305.22 | 6.11 | 4.13 - 8.26 |
|  | KGF | 17.38 | 2.71 - 51.89 | 4.20 | 1.89 - 5.72 |
|  | PlGF | 28.45 | 2.38 - 786.71 | 4.87 | 1.75 - 9.62 |
|  | VEGFR1 | 89.52 | 17.08 - 345.43 | 6.50 | 4.18 - 8.44 |
|  | VEGFR2 | 6618.36 | 3992.05  - 15432.1 | 12.69 | 11.96 - 13.91 |
|  | VEGF-D | 466.7 | 85.76 - 19578.63 | 8.86 | 6.44 - 14.25 |
|  | Ang1 | 2712.27 | 764.23 - 7029.95 | 11.40 | 9.58 - 12.77 |
|  | Tie2 | 17421.79 | 11102.86 - 40351.23 | 14.09 | 13.40 - 15.29 |
|  | Ang2 | 755.5 | 296.05 - 2035.69 | 9.55 | 8.21 - 10.99 |
|  | FGFb | 240.09 | 32.23 - 1978.94 | 7.91 | 4.99 - 10.94 |
|  | HGF | 366.93 | 151.54 - 887.68 | 8.52 | 7.25 - 9.79 |
|  | PDGFbb | 281.97 | 80.36 - 2262.45 | 8.14 | 6.35 - 11.12 |
|  | VEGF-A | 201.96 | 68.95 - 568.18 | 7.64 | 6.13 - 9.15 |
|  | VEGF-C | 376.29 | 40.46 - 1456.68 | 8.52 | 5.35 - 10.51 |
|  | SDF1b | 1729.9 | 258.37 - 3566.89 | 10.76 | 8.01 - 11.8 |
|  | E-selectin | 14216.63 | 5365.46 - 24011.62 | 13.78 | 12.35 - 14.54 |
|  | VCAM-1 | 915123.28 | 624503.98 - 2491890.24 | 19.80 | 19.23 - 21.25 |
|  | M65 | 1835.44 | 417.33  -2000 | 10.84 | 8.71 - 10.97 |
|  | CTCs | 3.5 | 0.5  - 17 | 2.08 | 0.50 - 4.17 |
| Imaging | iAUC (mmol.s) | 16.86 | 6.96 - 29.31 | 16.86 | 6.96 - 29.31 |
|  | *K*^trans^ (/min) | 0.14 | 0.06  - 0.26 | 13.51 | 5.99 - 25.73 |
|  | ν_e_ | 0.28 | 0.18  - 0.5 | 0.28 | 0.18 - 0.5 |
|  | ν_p_ | 0.01 | 0.004 - 0.03 | 0.01 | 0.004 - 0.03 |
|  | T1 (ms) | 1087.2 | 864.3 - 1940.31 | 10.09 | 9.76 - 10.92 |
|  | WTV (mm^3^) | 104246.45 | 18285.84 - 852514.95 | 16.67 | 14.15 - 19.7 |
|  | ETV (mm^3^) | 100512.01 | 18285.84 - 826966.29 | 16.62 | 14.15 - 19.66 |
|  | EF | 98.28 | 92.62 - 100 | 98.28 | 92.62 - 100 |
|  | ADC (x10^-3^mm2/s) | 1.03 | 0.91 - 1.63 | 0.10 | 0.09 - 0.16 |

***Table 4: Pre-treatment biomarkers associated with progression free survival***

Ang2 and VEGFA are significantly associated with progression free survival. The lower the concentration pre-treatment, the worse the progression free survival outcome. Both biomarkers are highly associated with each other and have very similar HR and p-values. No other pre-treatment biomarkers are associated with PFS.

Biomarker measurements were all log2 transformed, except ADC and *K*^trans^ which were multiplied by 100, and iAUC, ν_e_, ν_p_ and EF which were not changed.

All circulating biomarkers measured in pg/ml. Imaging biomarkers are stated. ν_e_, ν_p_ and EF have no units.

| **Biomarker type** | **Biomarker name** | **HR [95% CI]** | **p-value** |
| --- | --- | --- | --- |
| Circulating | Ang2 | 0.41 [0.19 - 0.86] | 0.019 |
|  | VEGF-A | 0.41 [0.19 - 0.87] | 0.021 |
|  | VEGF-C | 0.67 [0.44 - 1.04] | 0.075 |
|  | Ang1 | 0.51 [0.24 - 1.07] | 0.076 |
|  | E-selectin | 0.51 [0.23 - 1.10] | 0.084 |
|  | KGF | 0.62 [0.36 - 1.08] | 0.092 |
|  | HGF | 0.47 [0.19 - 1.18] | 0.108 |
|  | PDGFbb | 0.64 [0.34 - 1.18] | 0.151 |
|  | FGFb | 0.76 [0.52 - 1.12] | 0.166 |
|  | VEGFR1 | 0.75 [0.44 - 1.28] | 0.290 |
|  | IL8 | 0.83 [0.57 - 1.22] | 0.338 |
|  | PlGF | 0.89 [0.69 - 1.16] | 0.392 |
|  | VCAM-1 | 0.64 [0.23 - 1.78] | 0.393 |
|  | M65 | 0.83 [0.44 - 1.57] | 0.565 |
|  | VEGFR2 | 0.77 [0.31 - 1.93] | 0.581 |
|  | VEGF-D | 0.95 [0.76 - 1.18] | 0.614 |
|  | Tie2 | 0.82 [0.35 - 1.90] | 0.638 |
|  | CTCs | 1.09 [0.72 - 1.65] | 0.674 |
|  | IL6 | 0.96 [0.74 - 1.23] | 0.727 |
|  | SDF1b | 1.01 [0.56 - 1.81] | 0.972 |
| Imaging | ADC (x10^-3^mm2/s) | 3.56 [0.01 - 9.62] | 0.096 |
|  | ν_e_ | 49.44 [0.26 - 9428.96] | 0.145 |
|  | T1 (ms) | 3.58 [0.44 - 29.25] | 0.235 |
|  | *K*^trans^ (/min) | 1.04 [0.94 - 1.14] | 0.463 |
|  | iAUC (mmol.s) | 1.03 [0.94 - 1.13] | 0.492 |
|  | ν_p_ | 3.00 [0 - 1.26] | 0.599 |
|  | EF | 1.05 [0.78 - 1.42] | 0.730 |
|  | ETV (mm^3^) | 1.01 [0.75 - 1.37] | 0.937 |
|  | WTV (mm^3^) | 1.01 [0.76 - 1.34] | 0.975 |

***Table 5: Pre-treatment biomarkers associated with overall survival***

When using the cut-off of p <0.025 for significance, no pre-treatment biomarkers were associated with overall survival.

Biomarker measurements were all log2 transformed, except ADC and *K*^trans^ which were multiplied by 100, and iAUC, ν_e_, ν_p_ and EF which were not changed.

All circulating biomarkers measured in pg/ml. Imaging biomarkers are stated. ν_e_, ν_p_ and EF have no units.

| **Biomarker type** | **Biomarker name** | **HR [95% CI]** | **p-value** |
| --- | --- | --- | --- |
| Circulating | M65 | 2.26 [0.99 - 5.16] | 0.053 |
|  | VEGF-C | 0.66 [0.42 - 1.05] | 0.078 |
|  | IL6 | 1.26 [0.96 - 1.67] | 0.098 |
|  | VCAM-1 | 1.90 [0.84 - 4.31] | 0.124 |
|  | PDGFbb | 0.58 [0.28 - 1.19] | 0.135 |
|  | FGFb | 0.76 [0.52 - 1.09] | 0.136 |
|  | CTCs | 1.35 [0.89 - 2.05] | 0.165 |
|  | IL8 | 1.31 [0.88 - 1.96] | 0.188 |
|  | PlGF | 1.15 [0.89 - 1.49] | 0.300 |
|  | Tie2 | 0.65 [0.28 - 1.53] | 0.321 |
|  | VEGF-D | 1.10 [0.86 - 1.41] | 0.436 |
|  | HGF | 0.76 [0.33 - 1.75] | 0.514 |
|  | Ang2 | 0.82 [0.43 - 1.57] | 0.553 |
|  | VEGF-A | 0.82 [0.40 - 1.67] | 0.575 |
|  | Ang1 | 0.84 [0.41 - 1.76] | 0.648 |
|  | SDF1b | 1.13 [0.64 - 1.98] | 0.682 |
|  | KGF | 1.11 [0.63 - 1.93] | 0.726 |
|  | VEGFR2 | 0.86 [0.36 - 2.05] | 0.729 |
|  | VEGFR1 | 1.04 [0.58 - 1.85] | 0.906 |
|  | E-selectin | 1.01 [0.47 - 2.17] | 0.982 |
| Imaging | T1 (ms) | 7.73 [1.15 - 51.83] | 0.035 |
|  | *K*^trans^ (/min) | 0.91 [0.84 – 1.00] | 0.048 |
|  | iAUC (mmol.s) | 0.94 [0.87 - 1.01] | 0.088 |
|  | ADC (x10^-3^mm2/s) | 4.54 [5.29 - 3.89] | 0.174 |
|  | ν_p_ | 2.13 [1.24 - 3.67] | 0.355 |
|  | EF | 0.88 [0.66 - 1.18] | 0.407 |
|  | ν_e_ | 0.19 [0.002, 19.43] | 0.486 |
|  | ETV (mm^3^) | 1.05 [0.78 - 1.42] | 0.759 |
|  | WTV (mm^3^) | 1.04 [0.78 - 1.37] | 0.814 |

**Biomarkers on treatment**

***Figure 1: Percentage change in circulating biomarkers on treatment***

X axis: Time points - 0 = mean pre-treatment concentration, 1 = Cycle 1 Day 2, 2 = Cycle 1 day 8, 3 = Cycle 2 day 2, 4 = 3 months, 5 = cycle 12

Y axis: percentage change from pre-treatment concentration


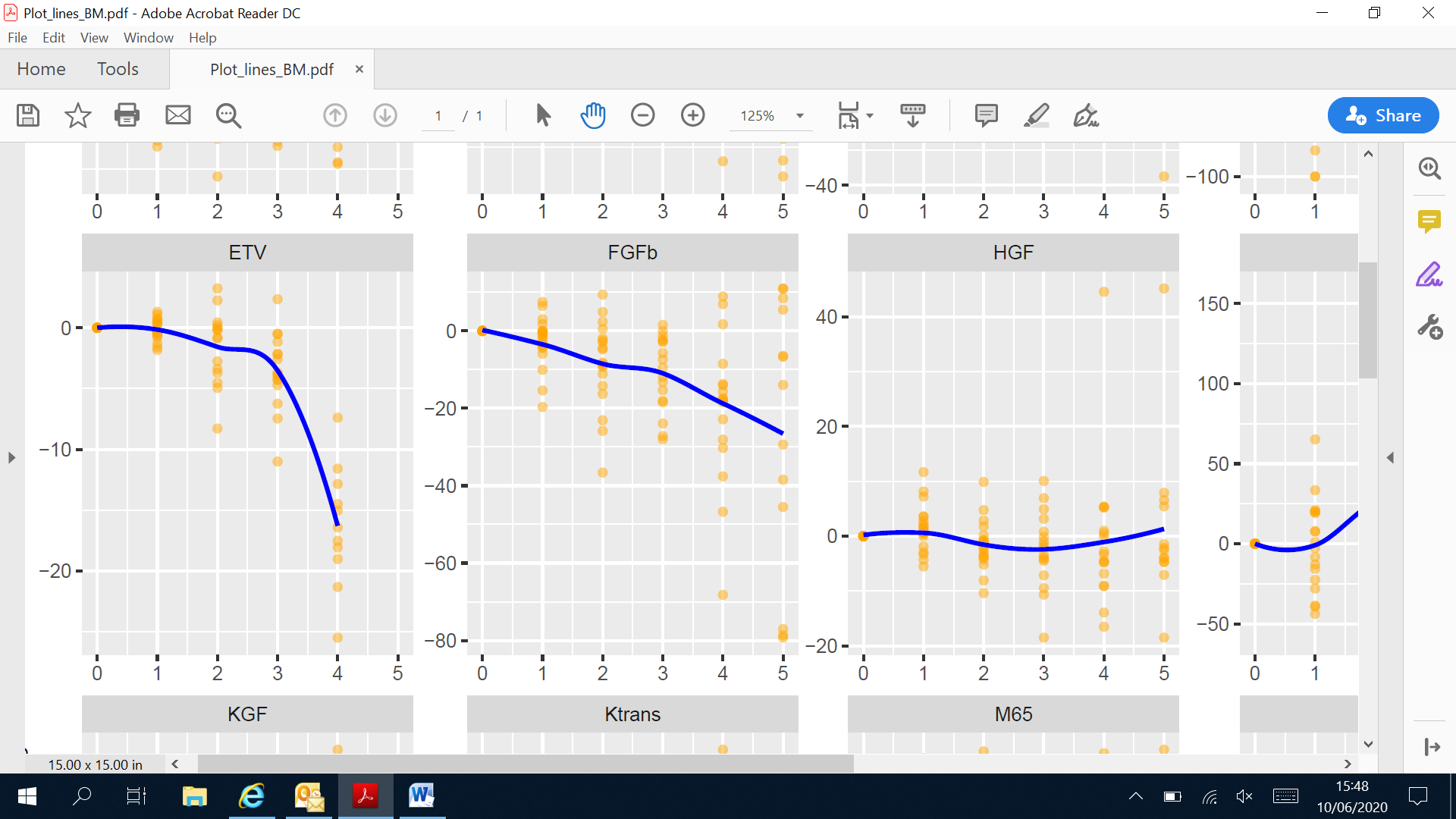

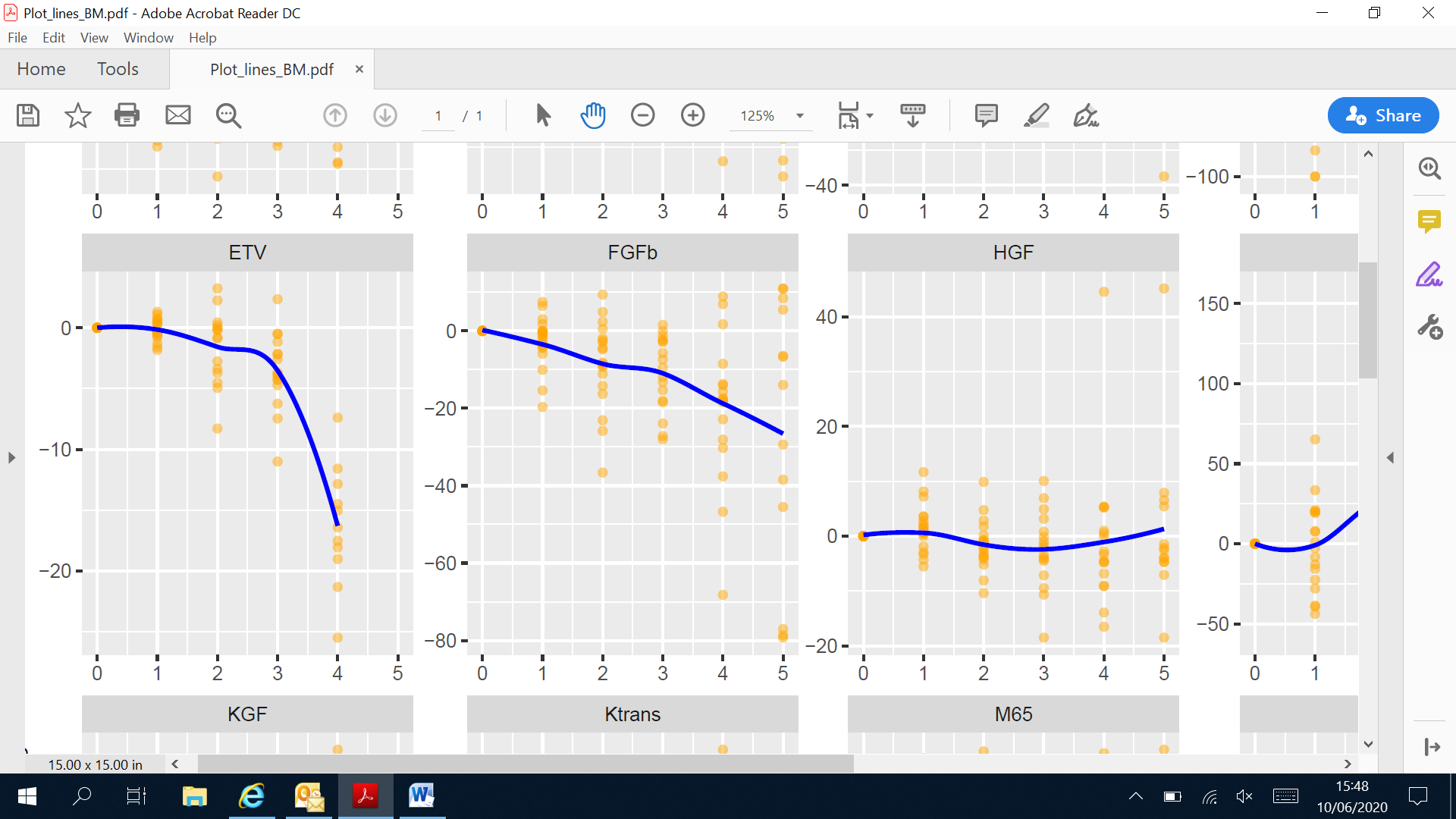

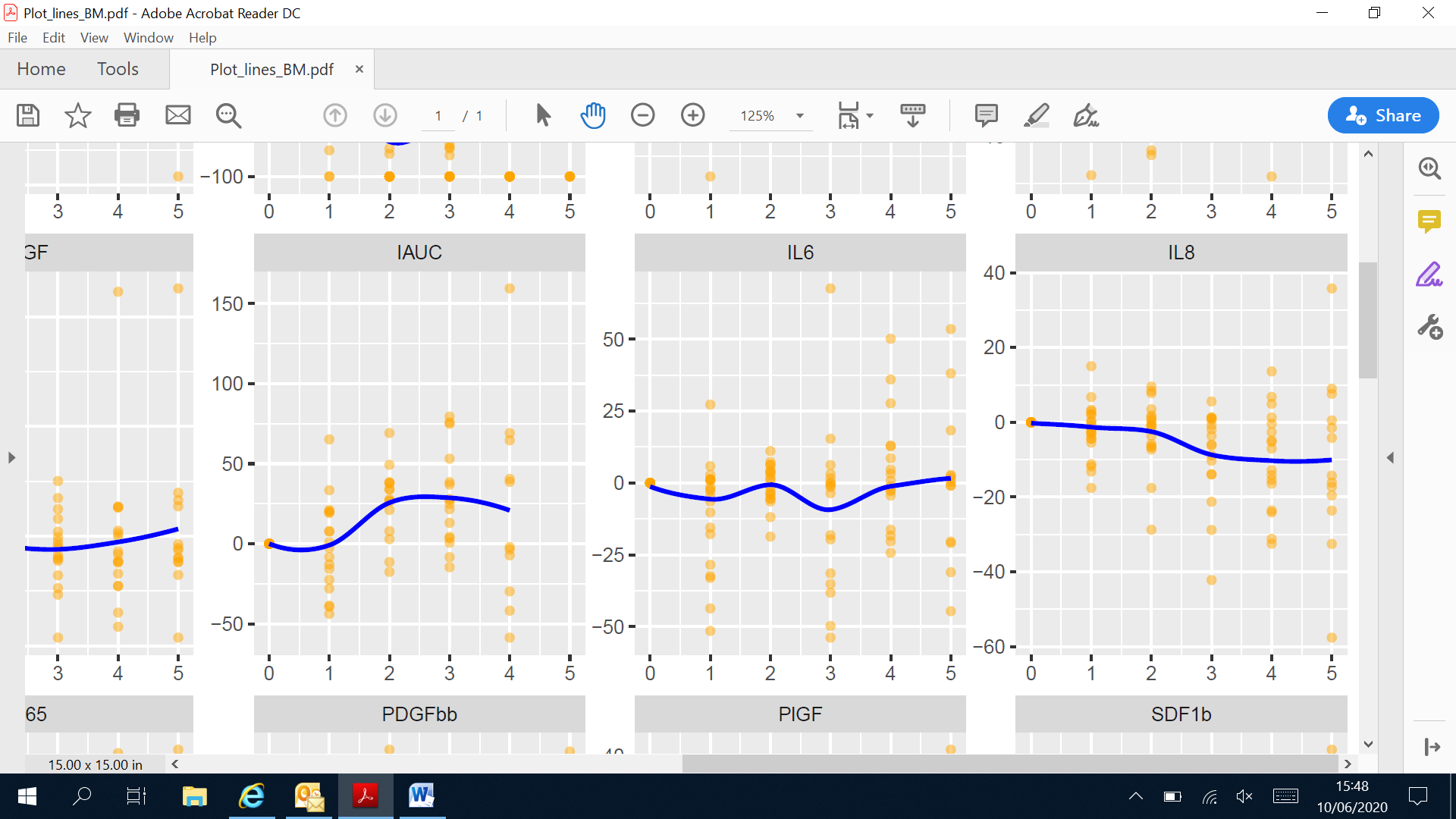

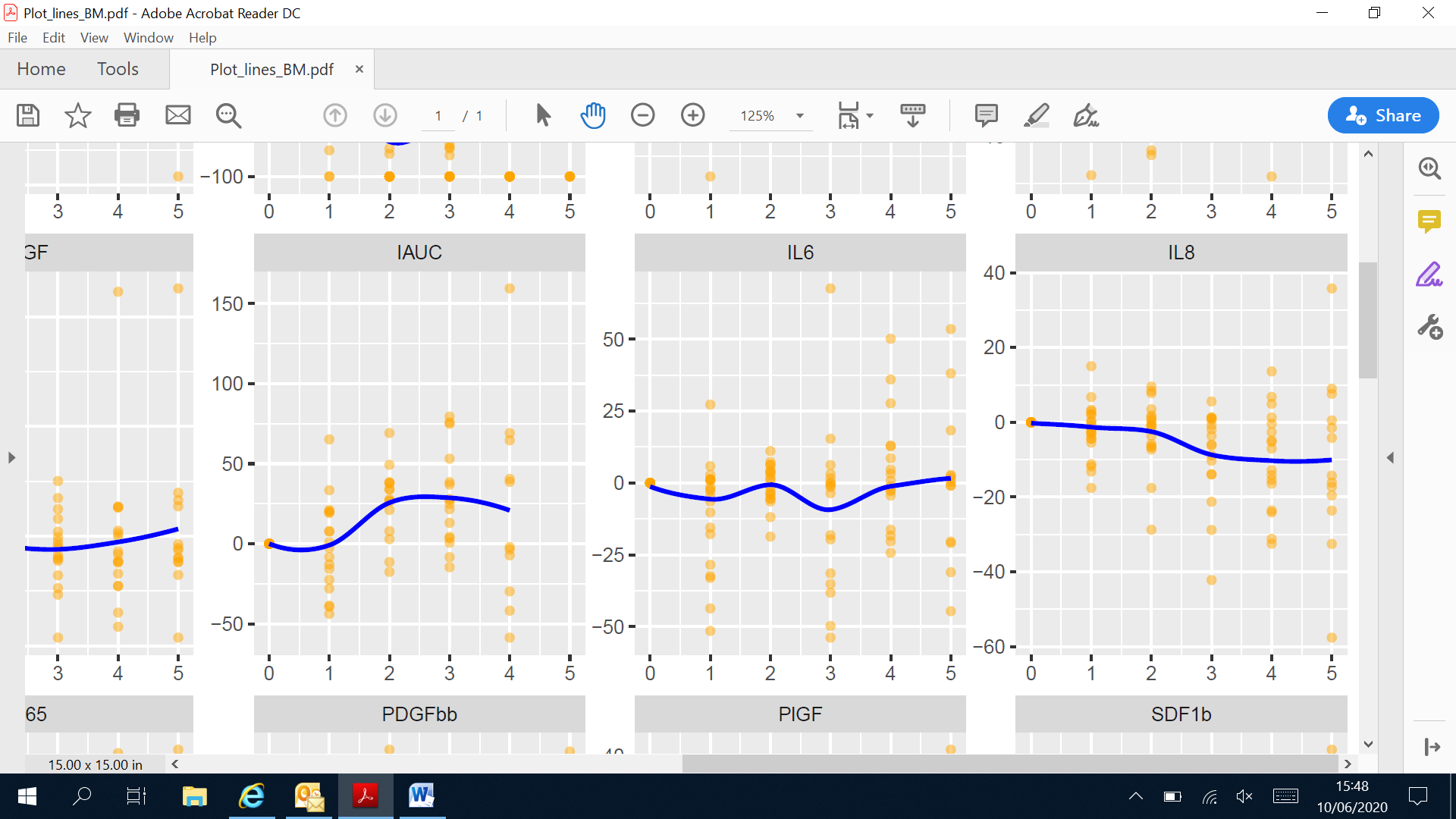

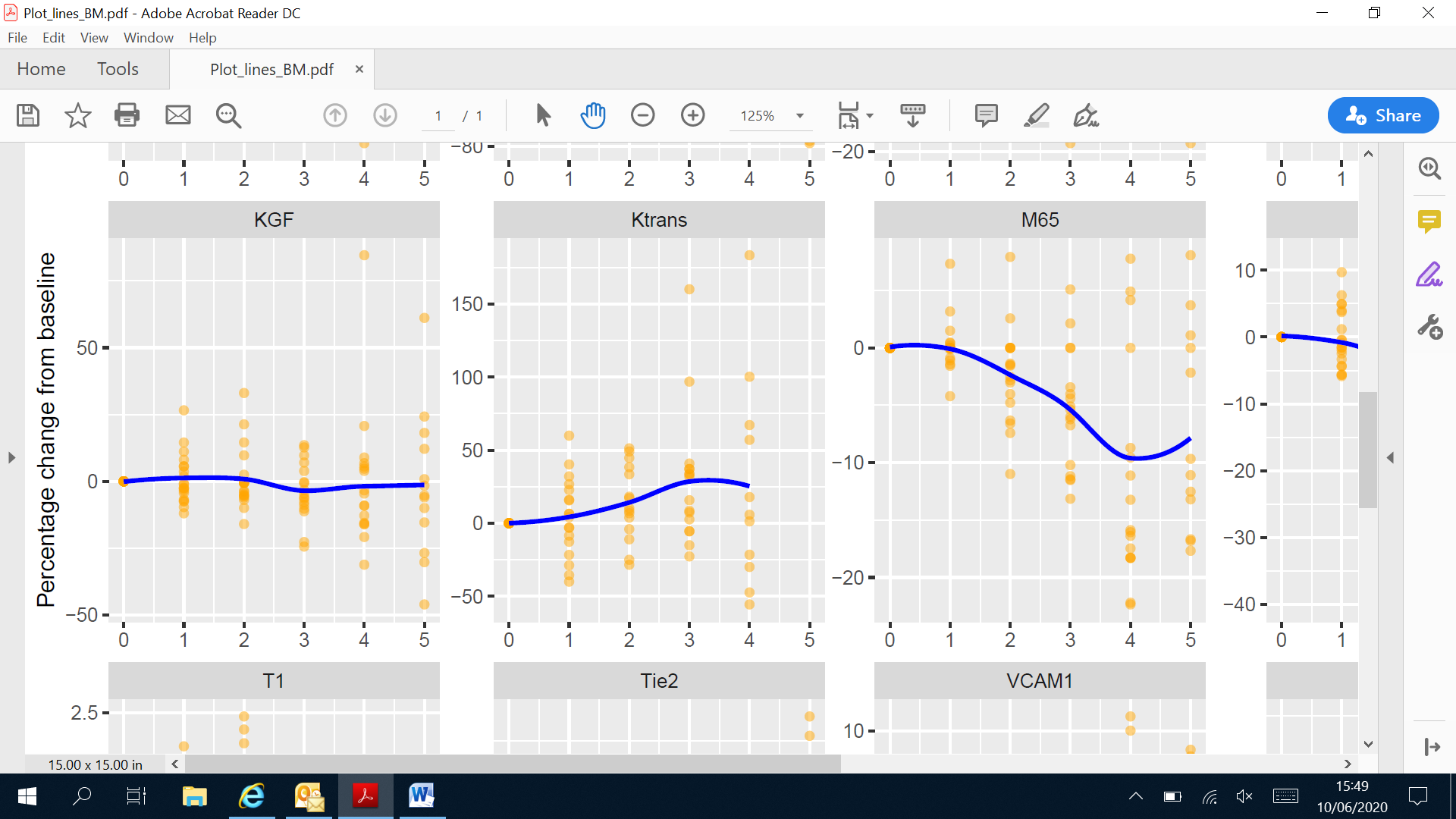

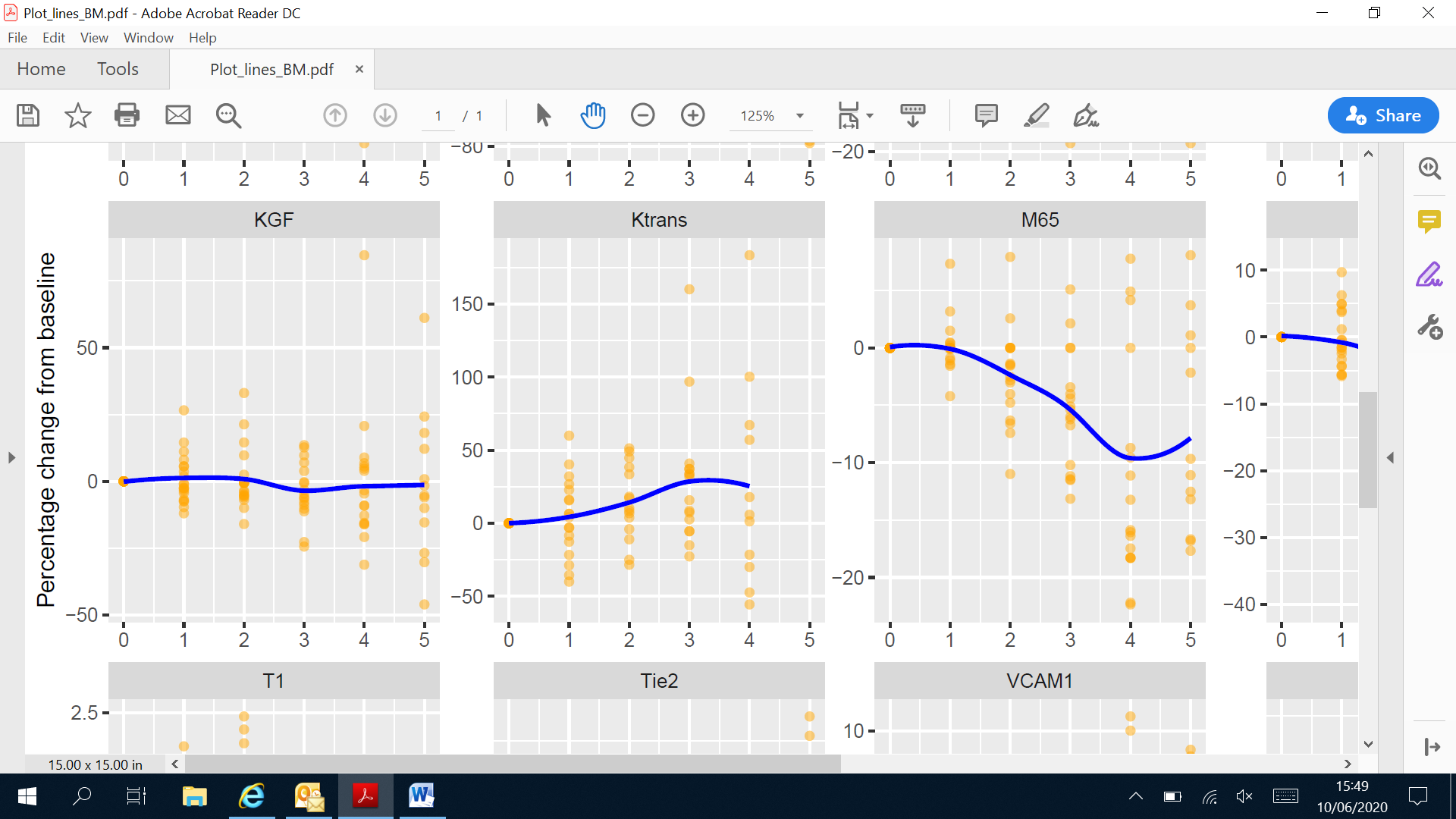

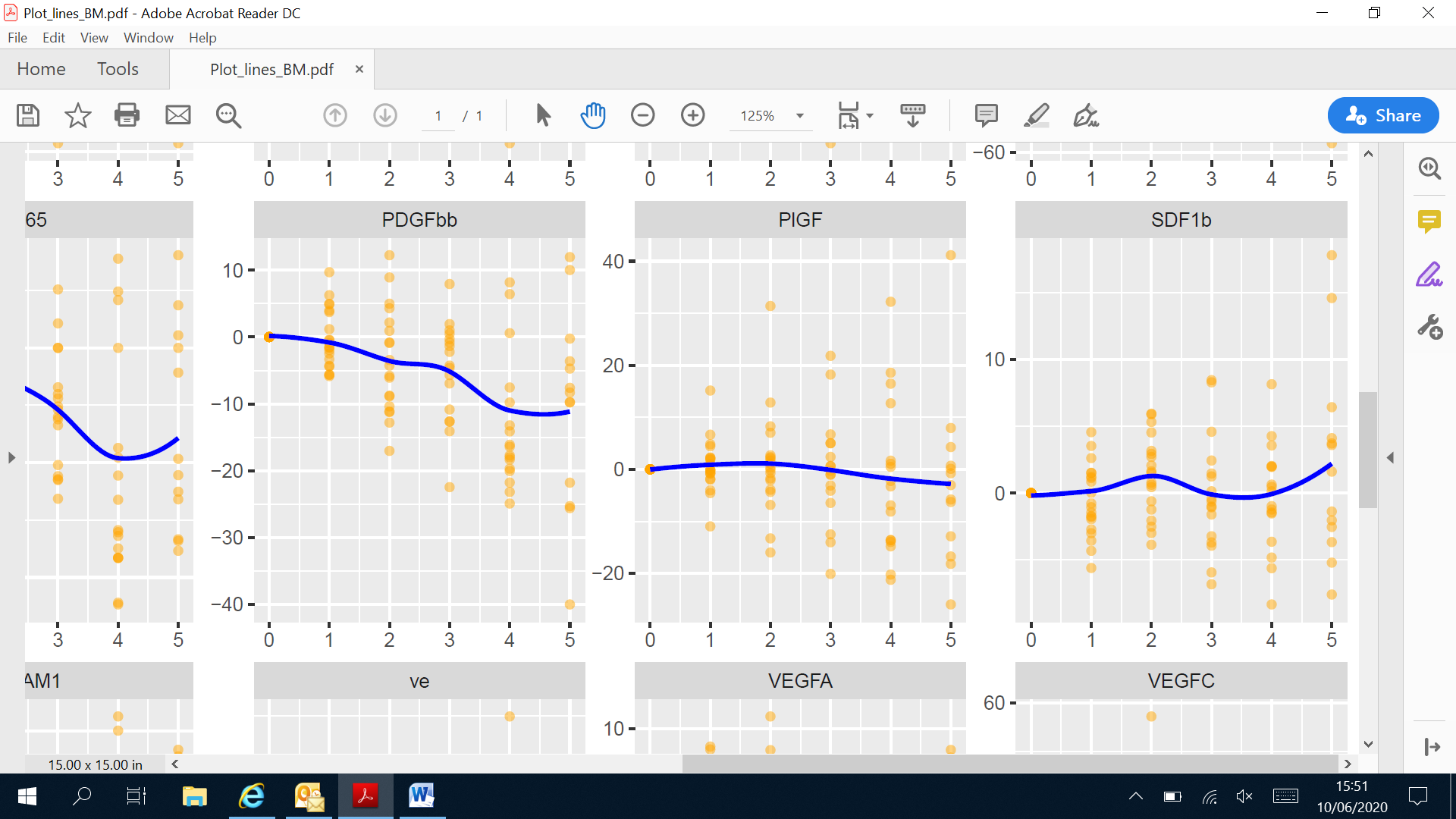

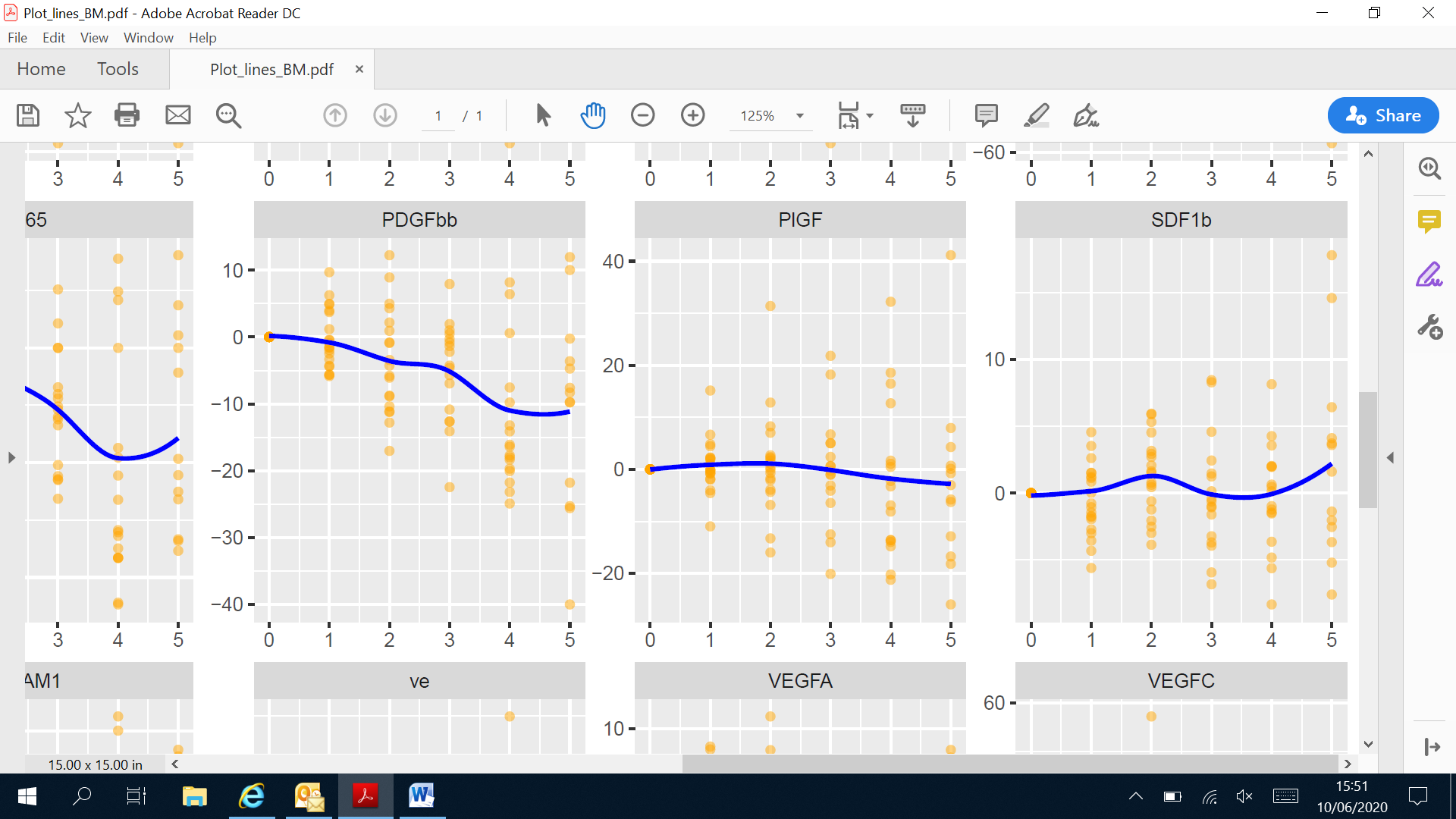

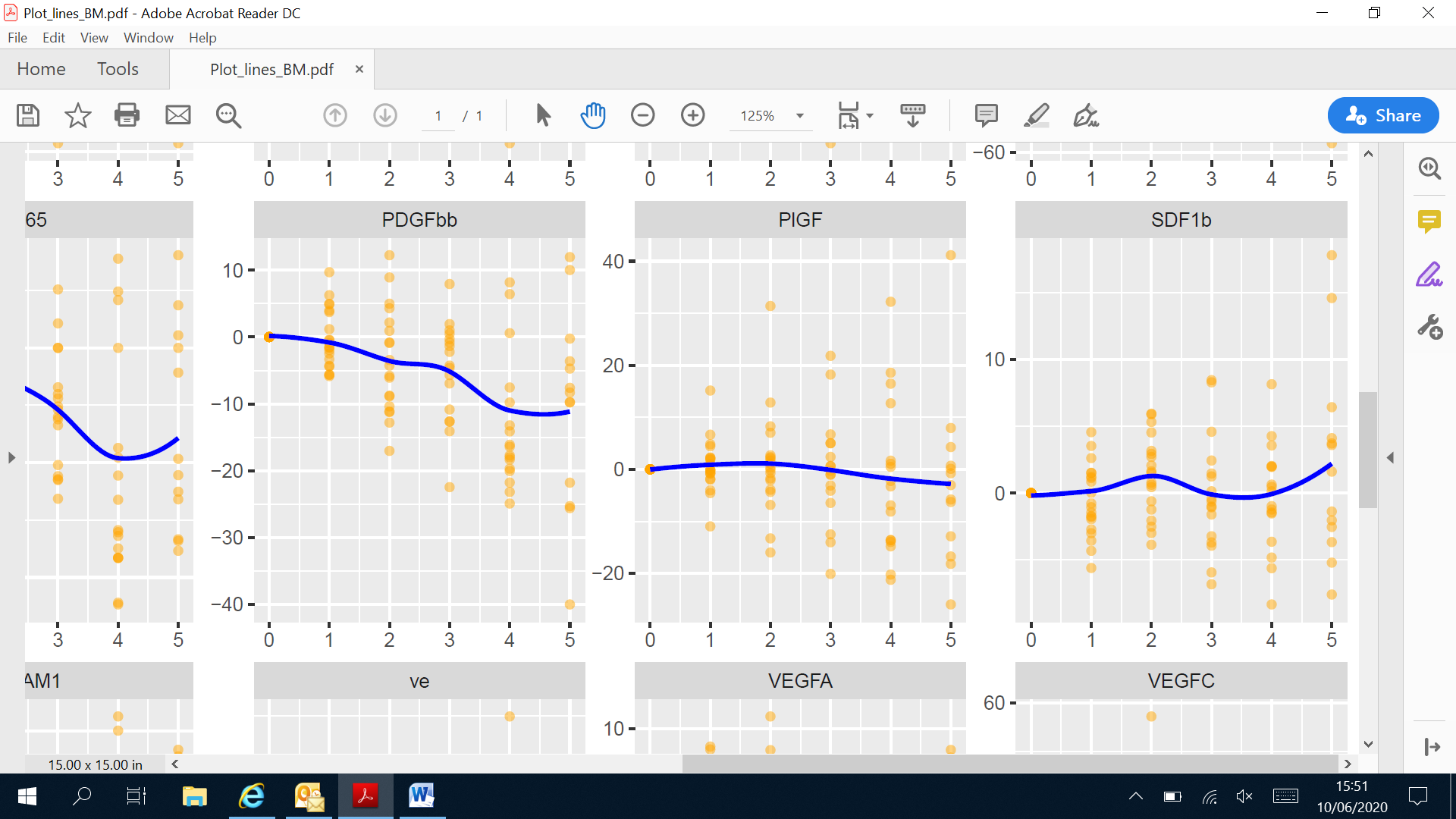

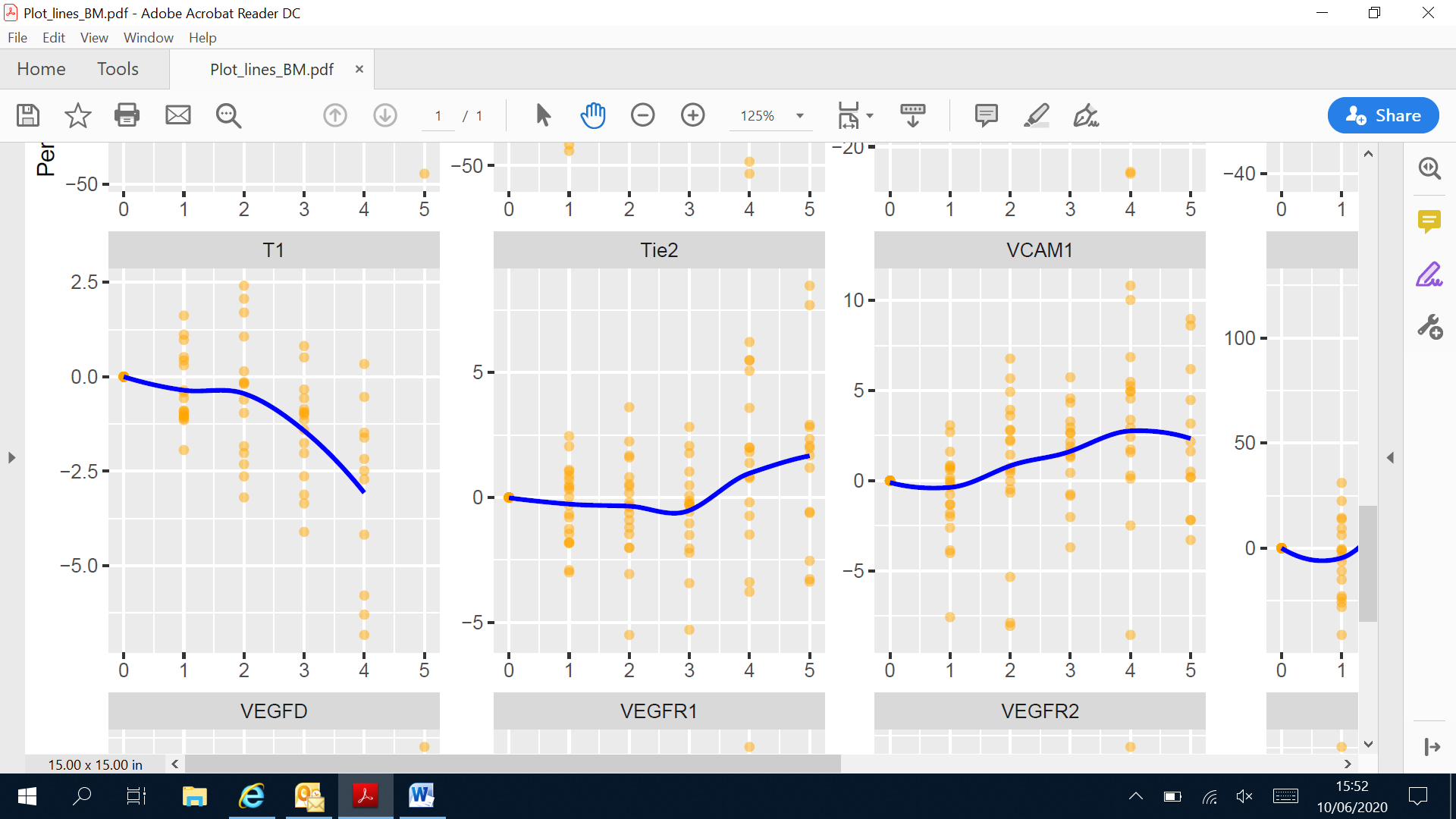

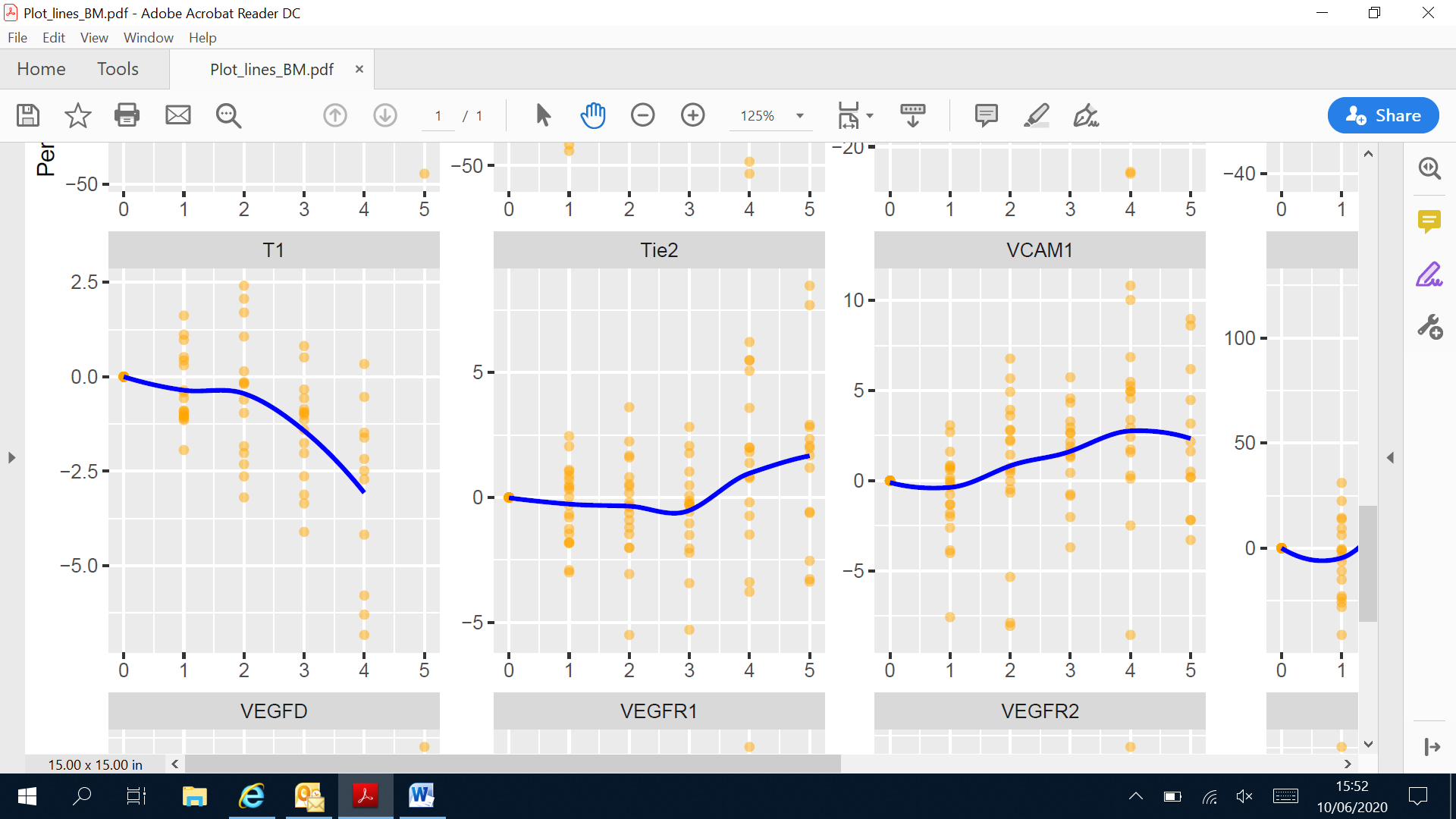

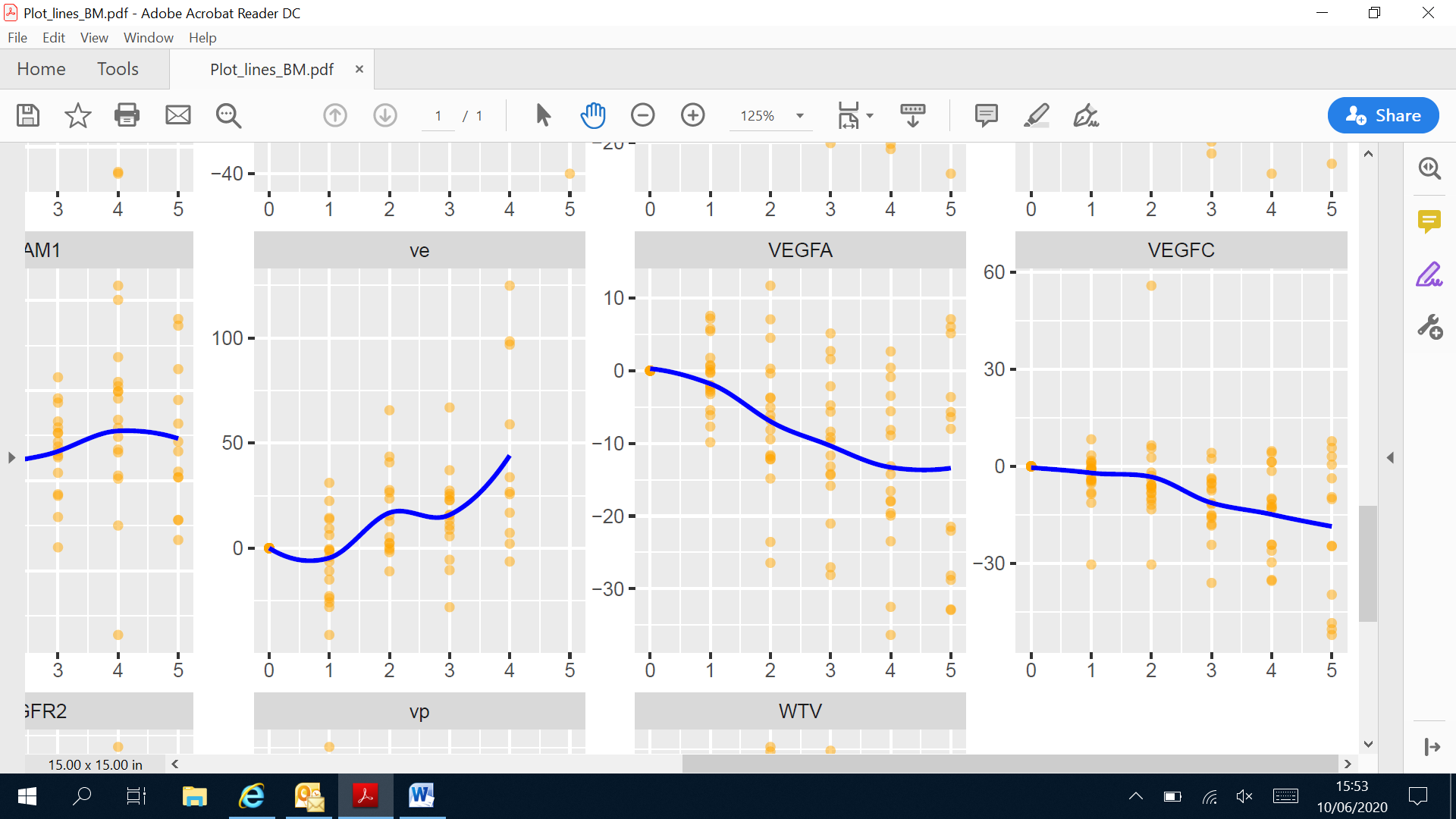

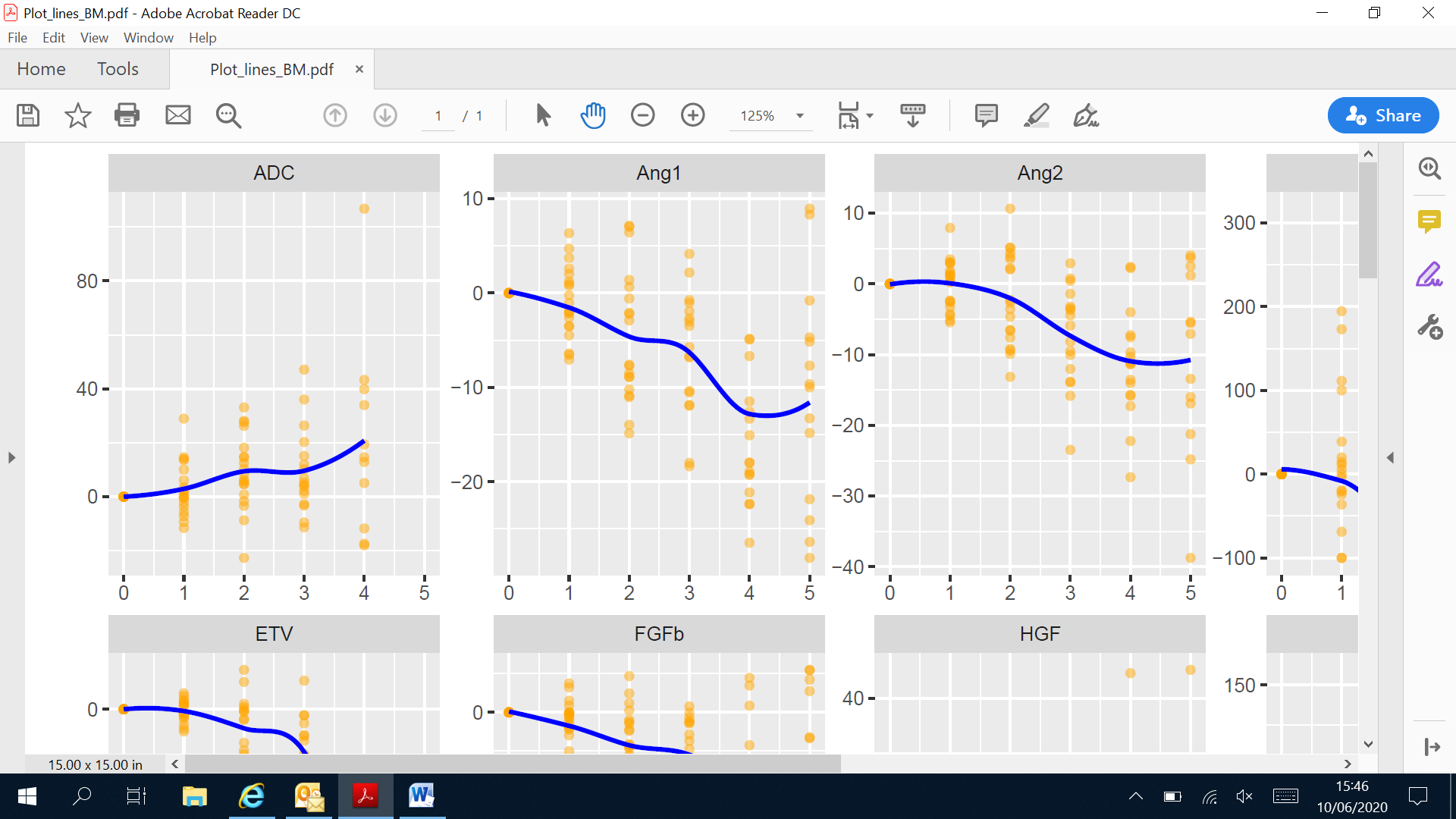

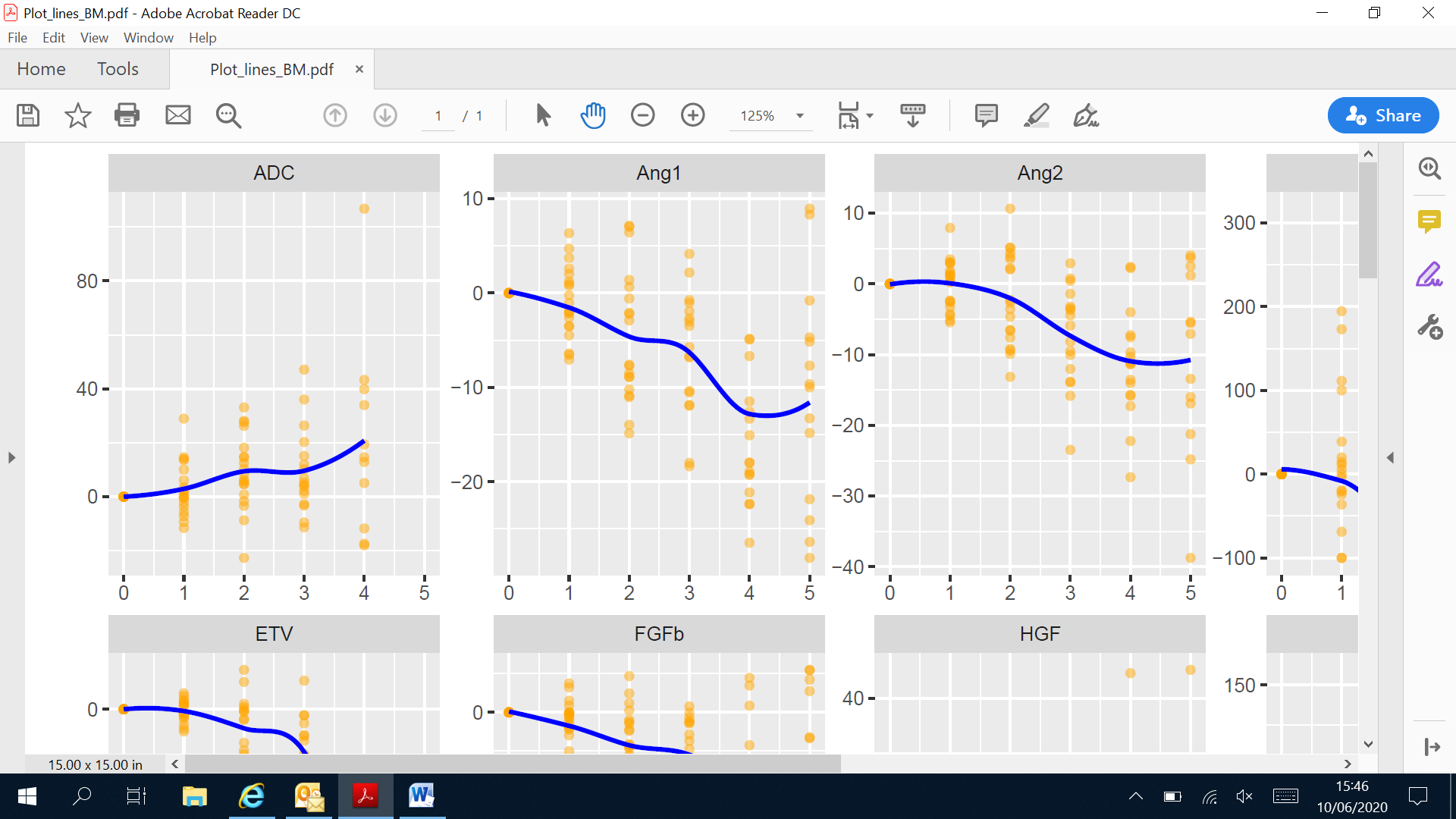

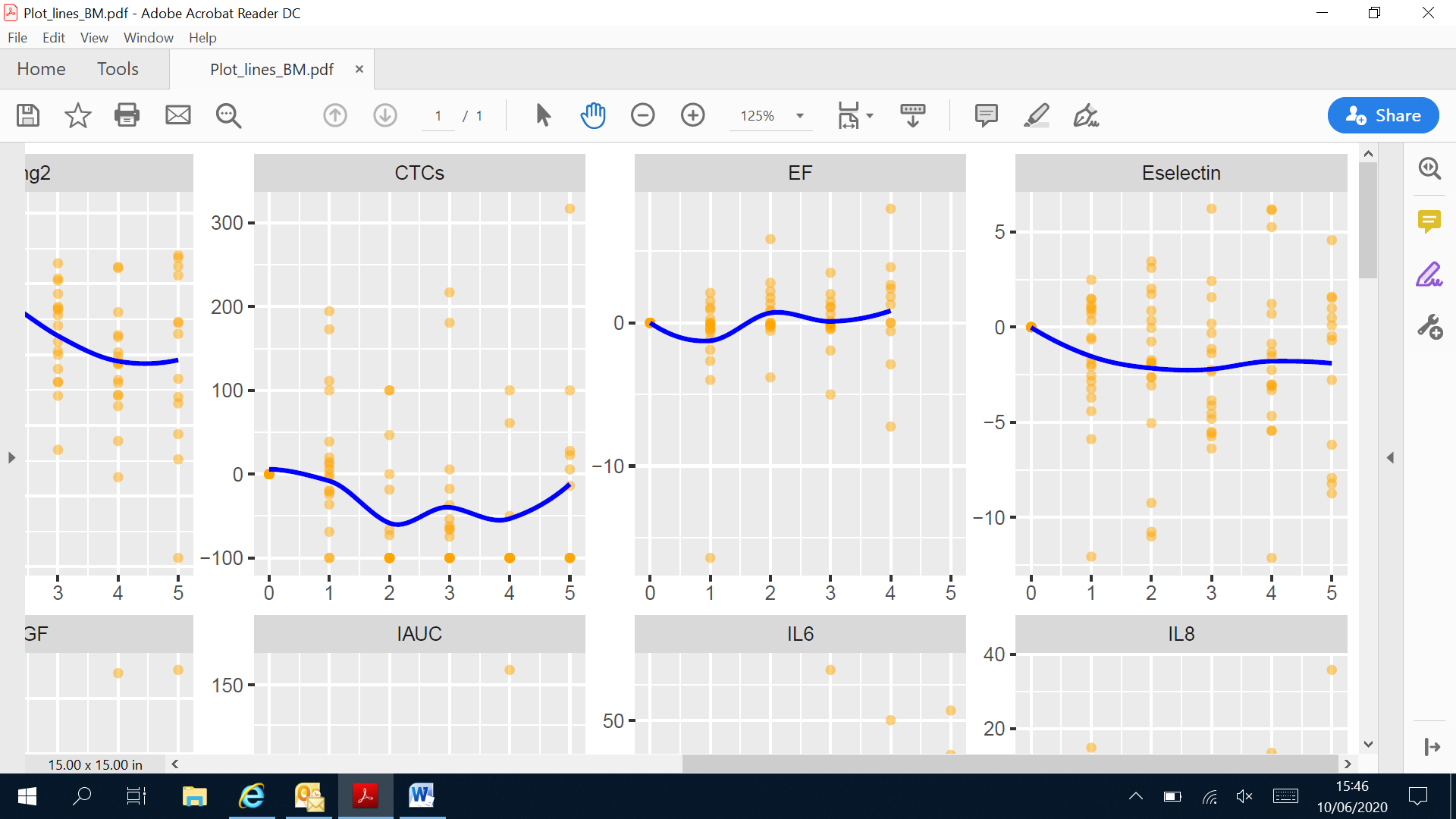

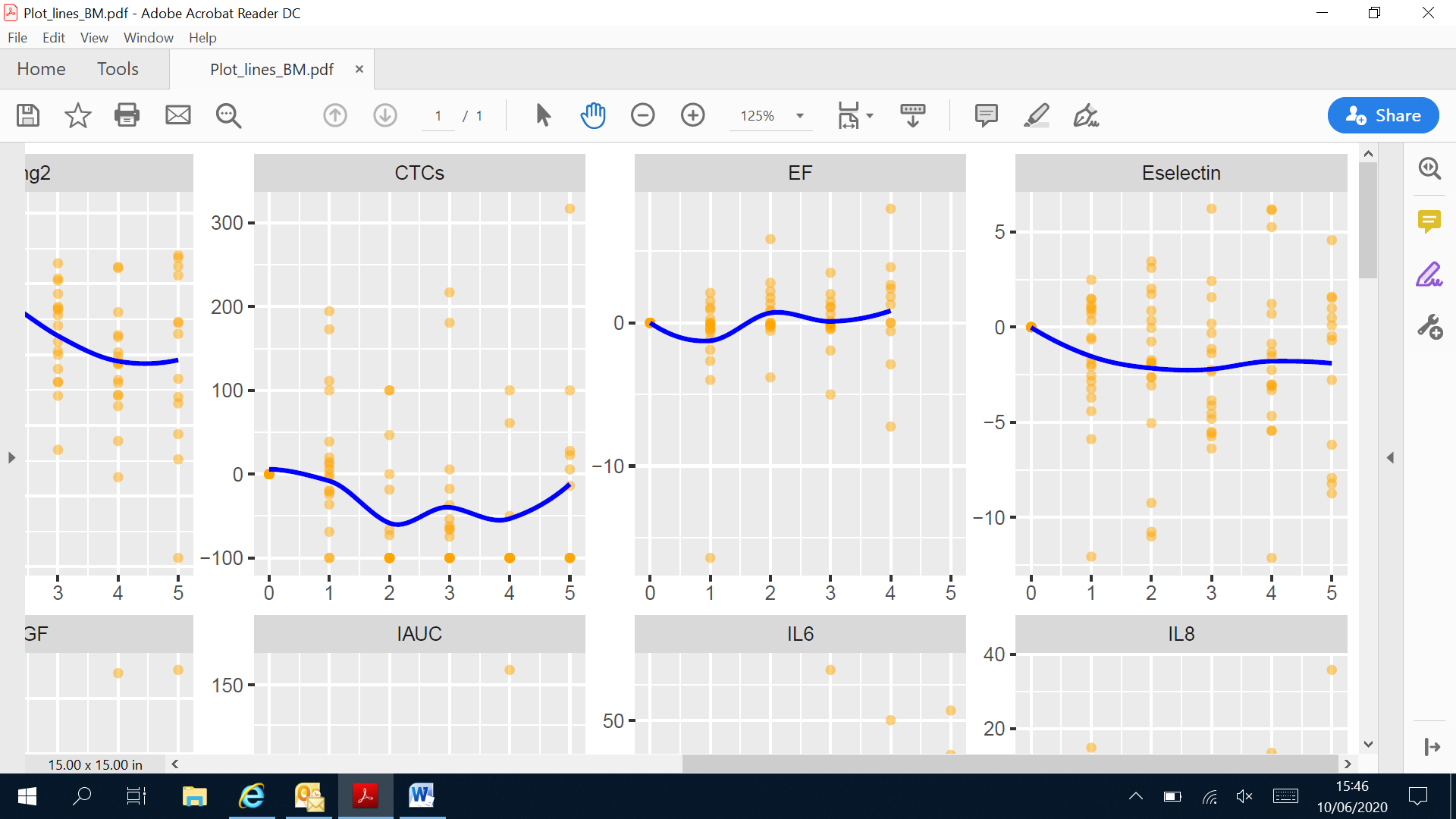


Ang1

Ang2

CTCs

E-selectin

FGFb

HGF

IL6

IL8

KGF

M65

PGDFbb

PlGF

SDF1b

Tie2

VCAM-1

VEGF-A


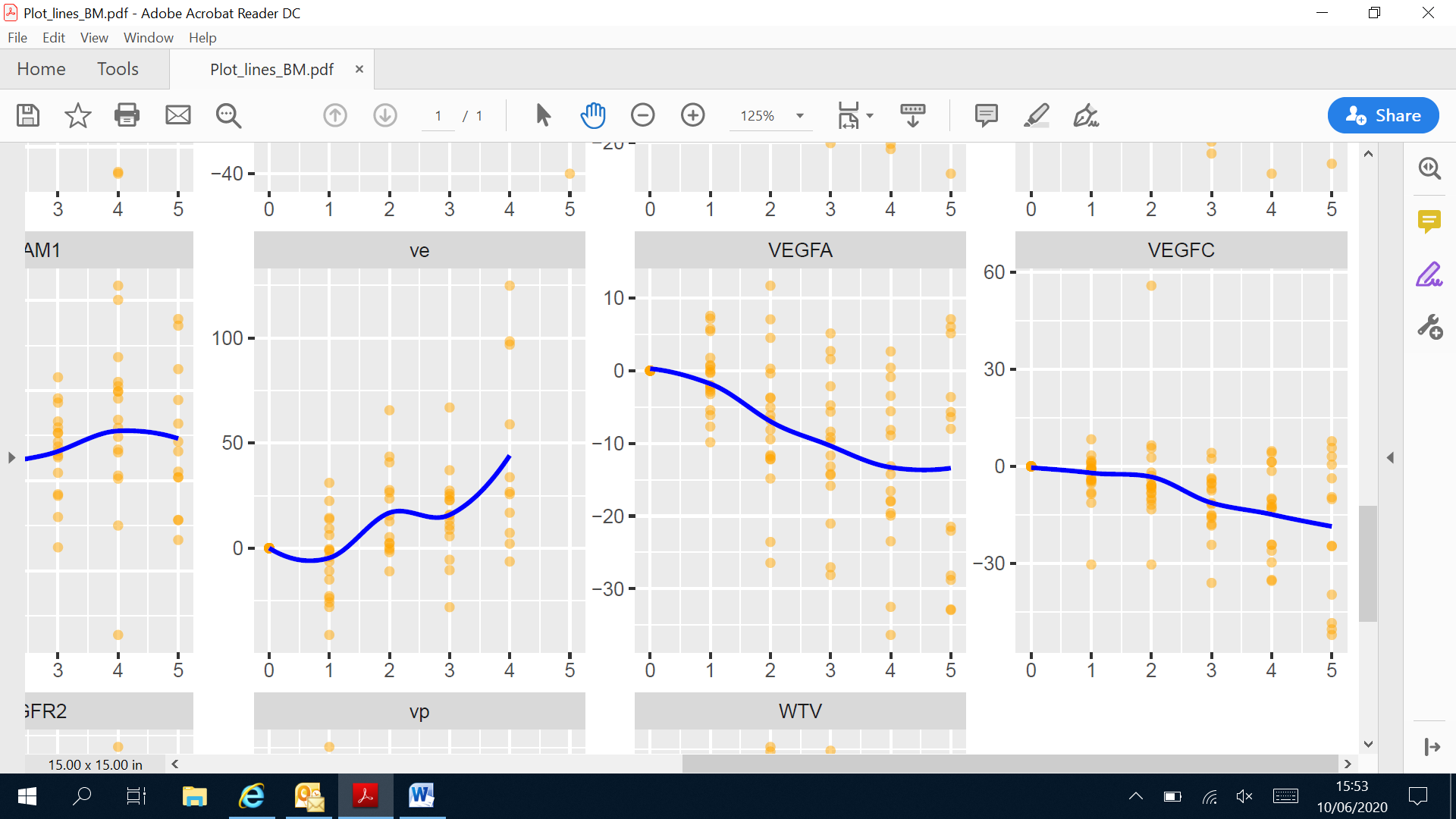

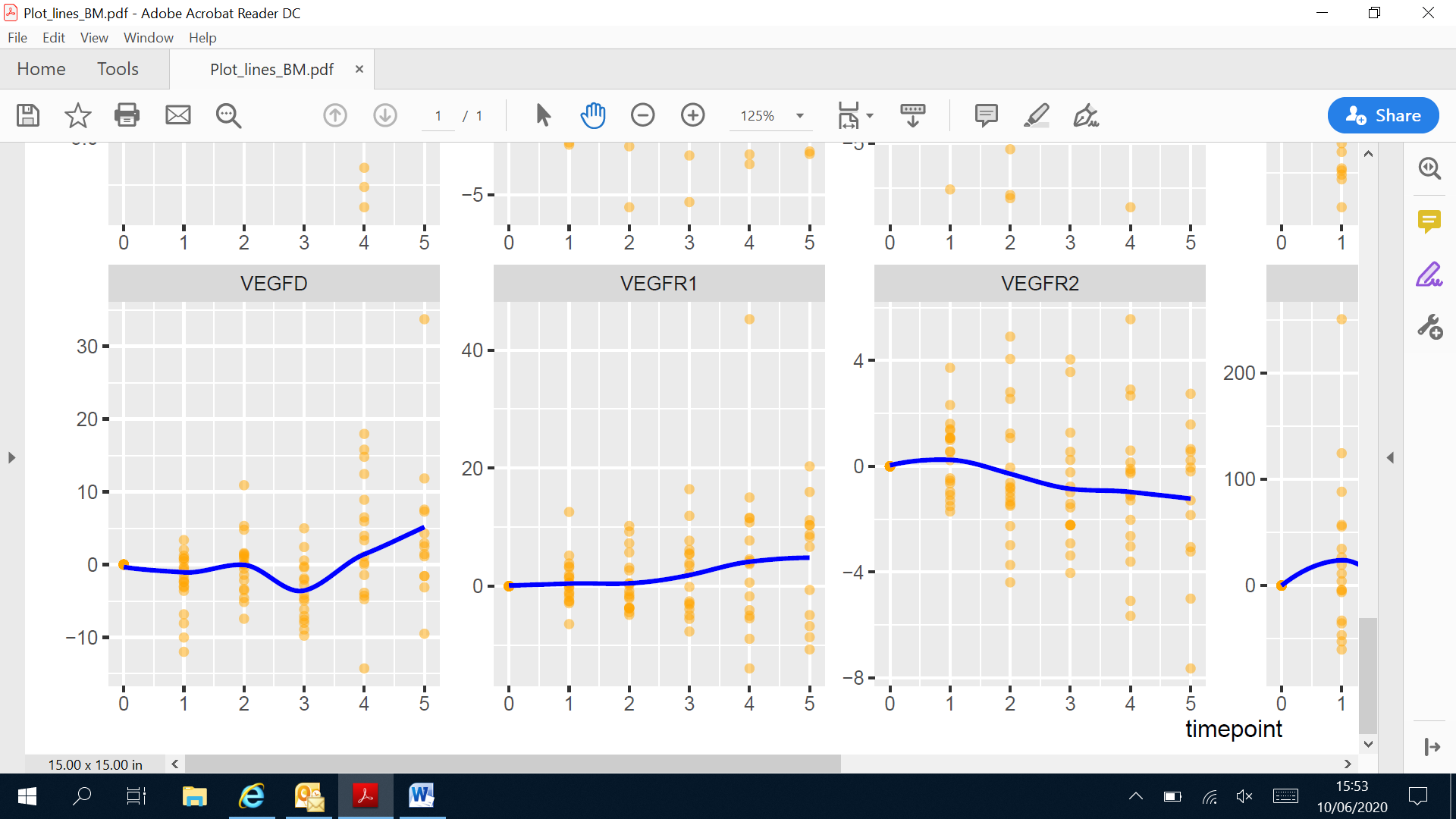

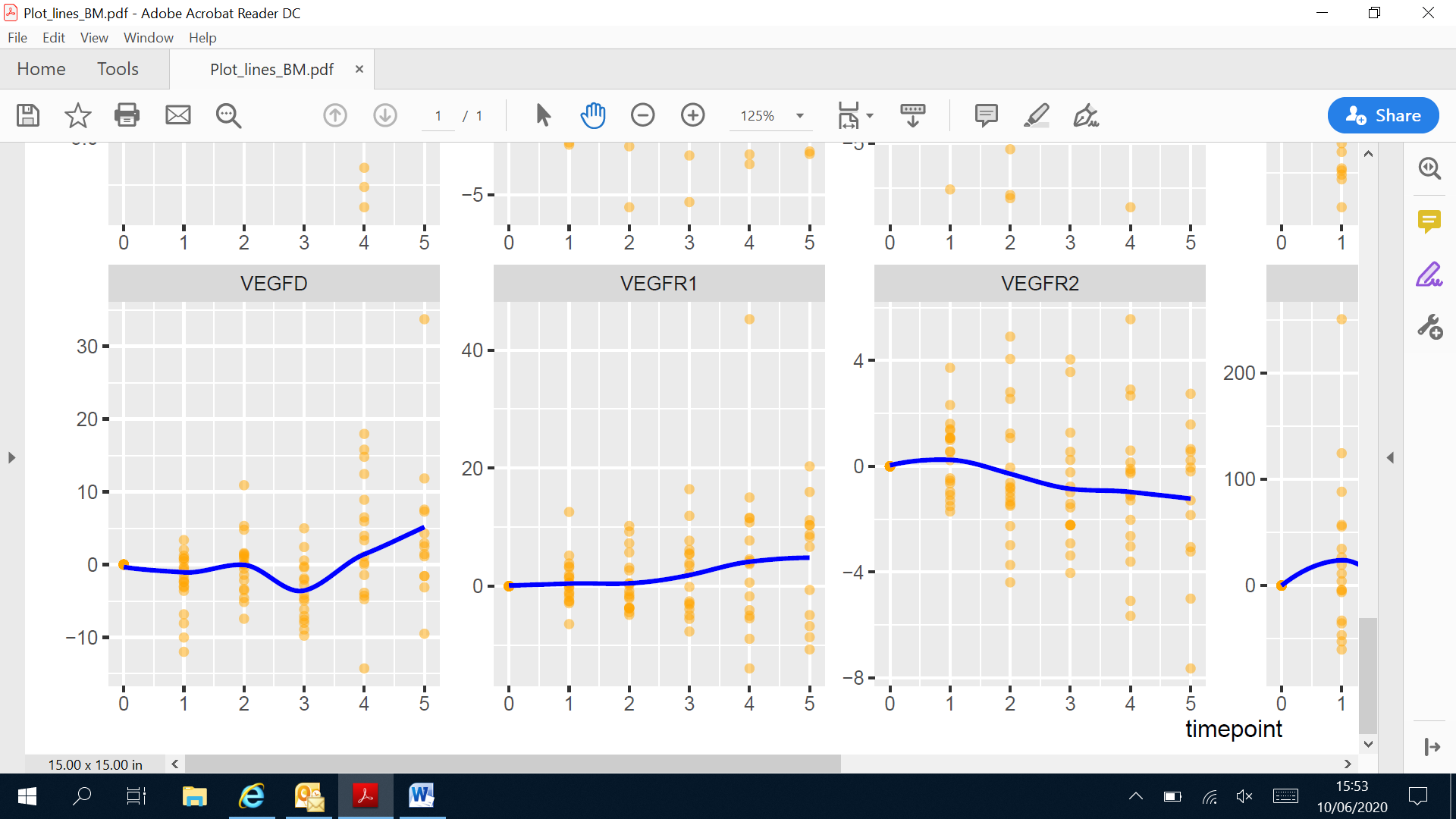

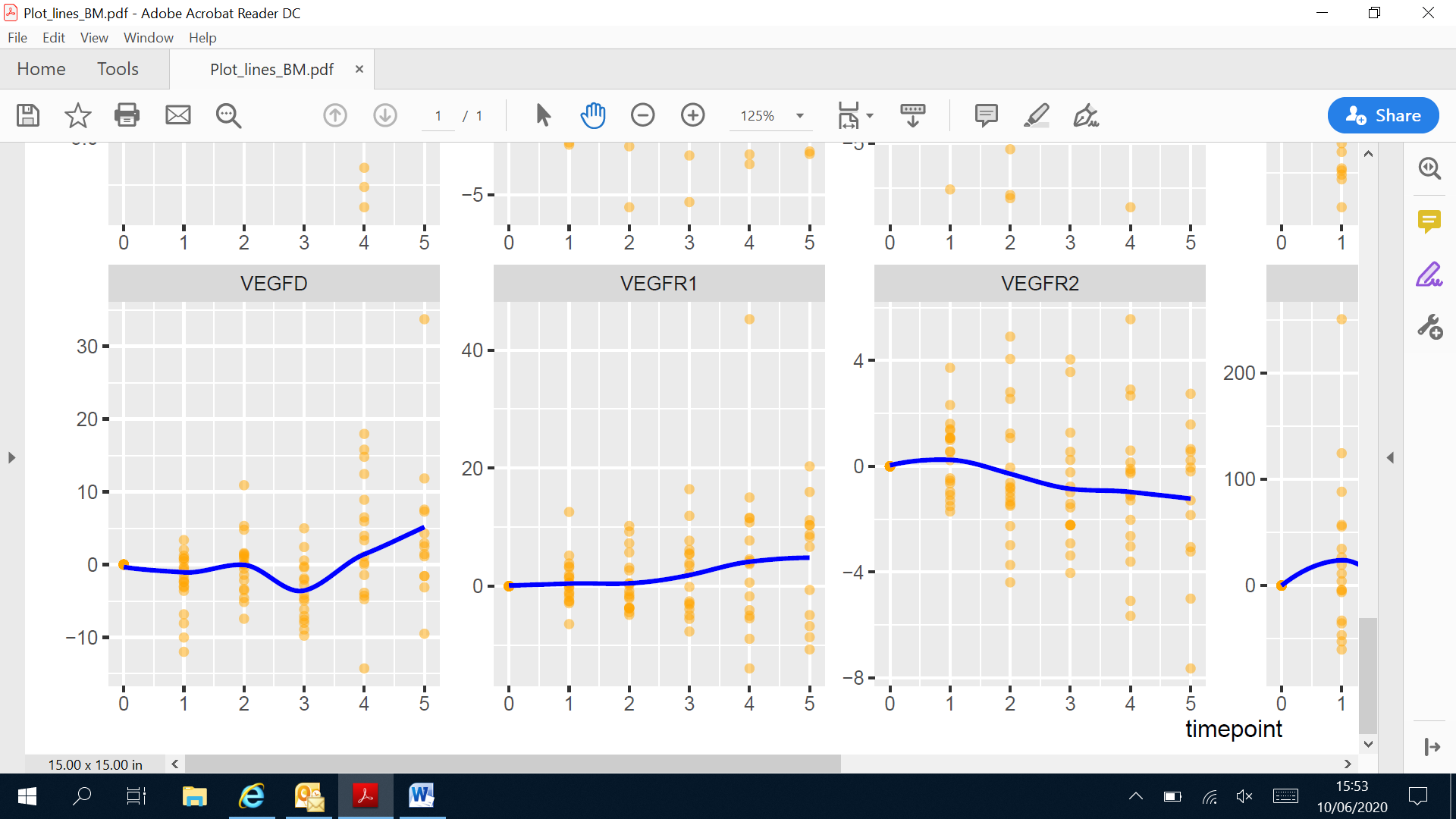


VEGF-C

VEGF-D

VEGFR1

VEGFR2

***Figure 2: Percentage change in imaging biomarkers on treatment***

X axis: Time points - 0 = mean pre-treatment concentration, 1 = Cycle 1 Day 2, 2 = Cycle 1 day 8, 3 = Cycle 2 day 2, 4 = 3 months

Y axis: percentage change from pre-treatment value


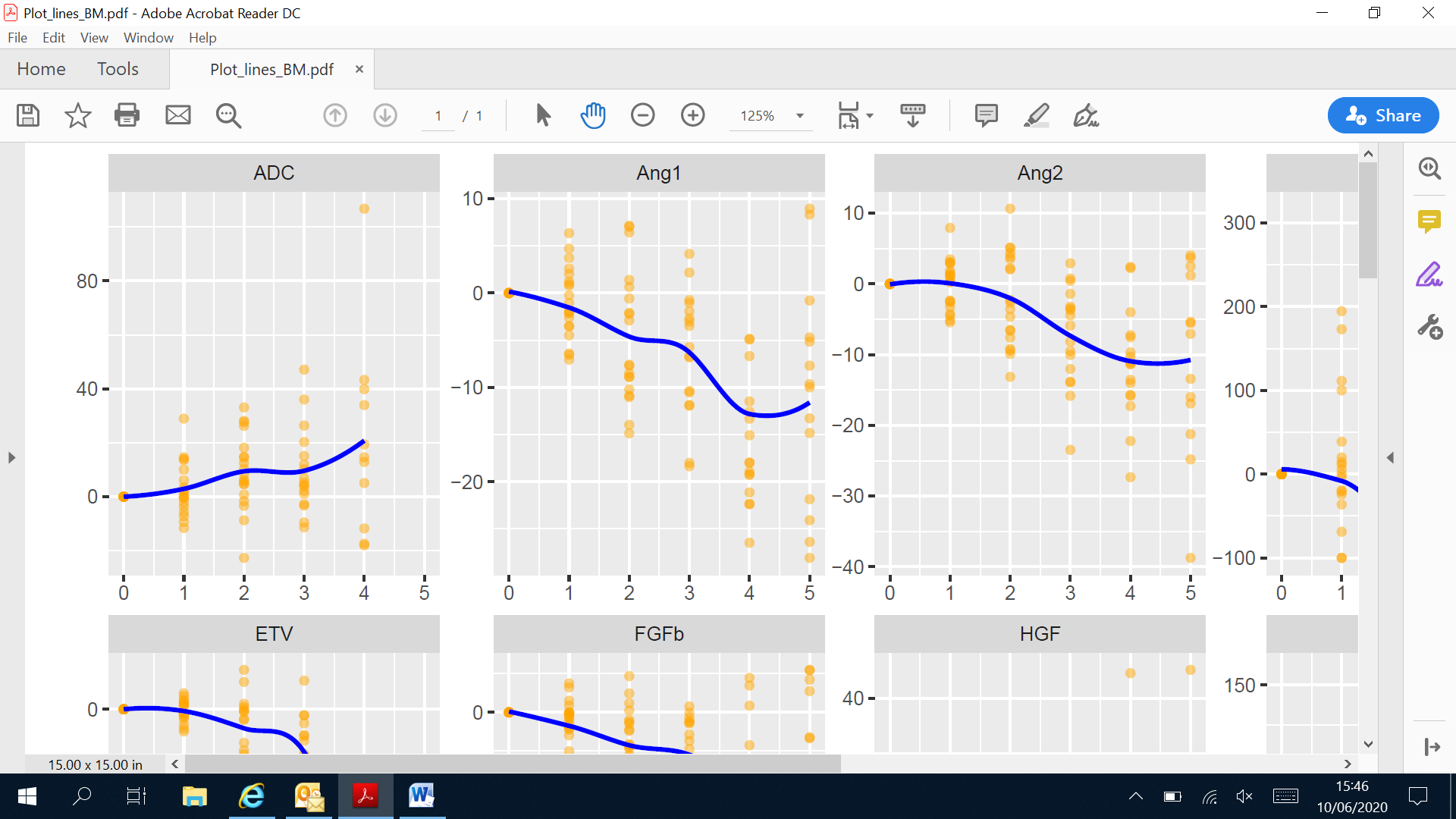

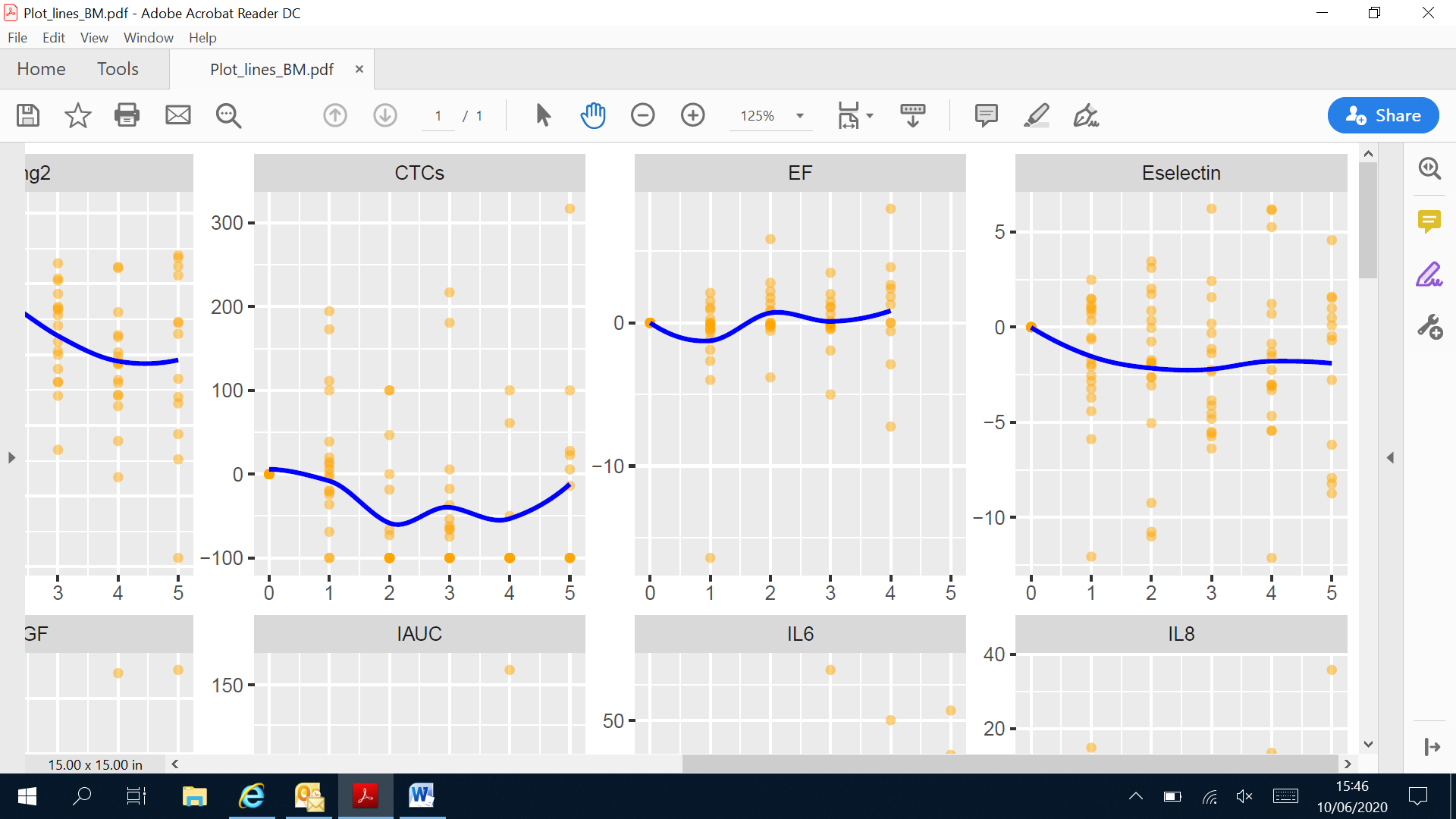

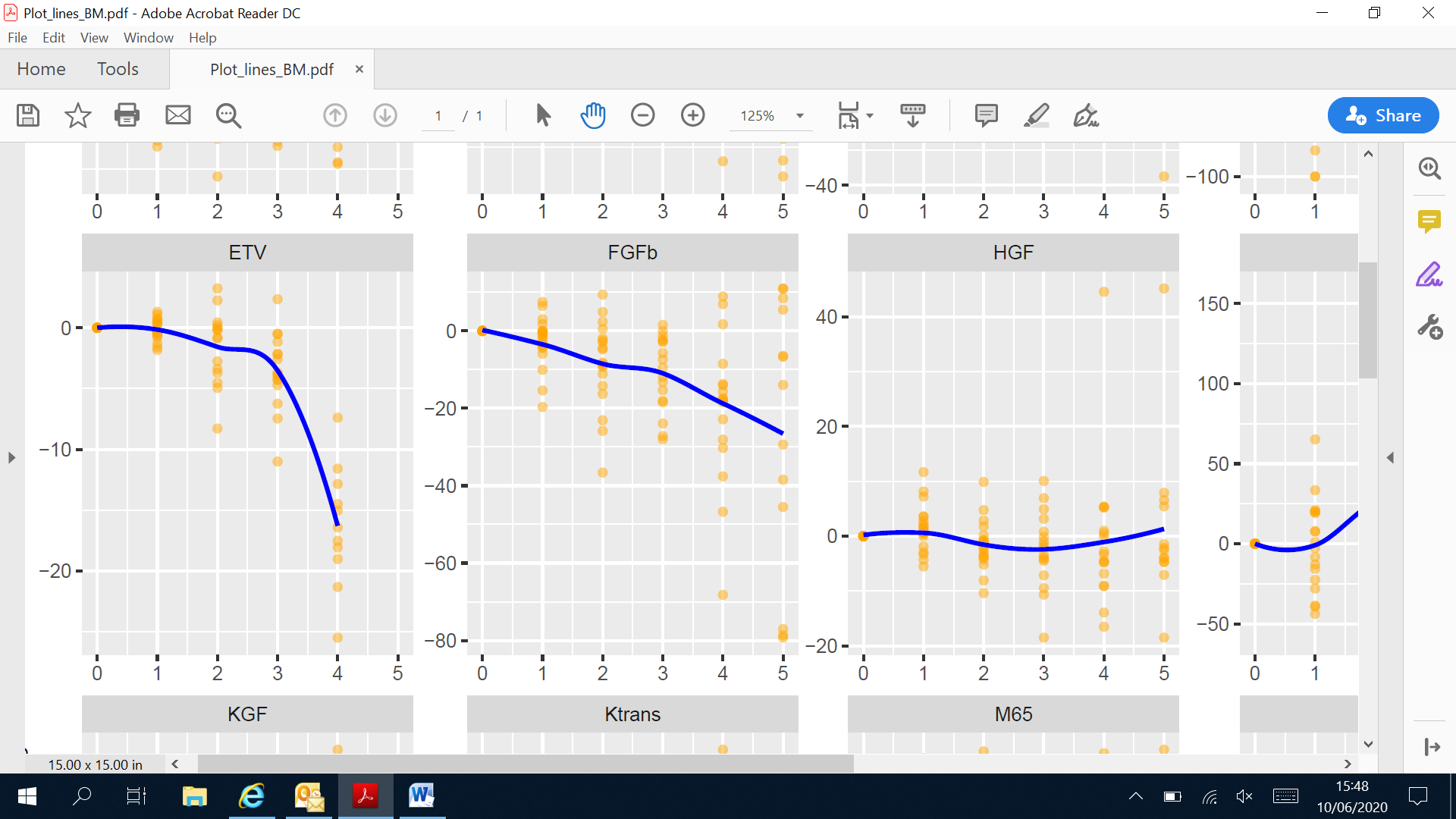

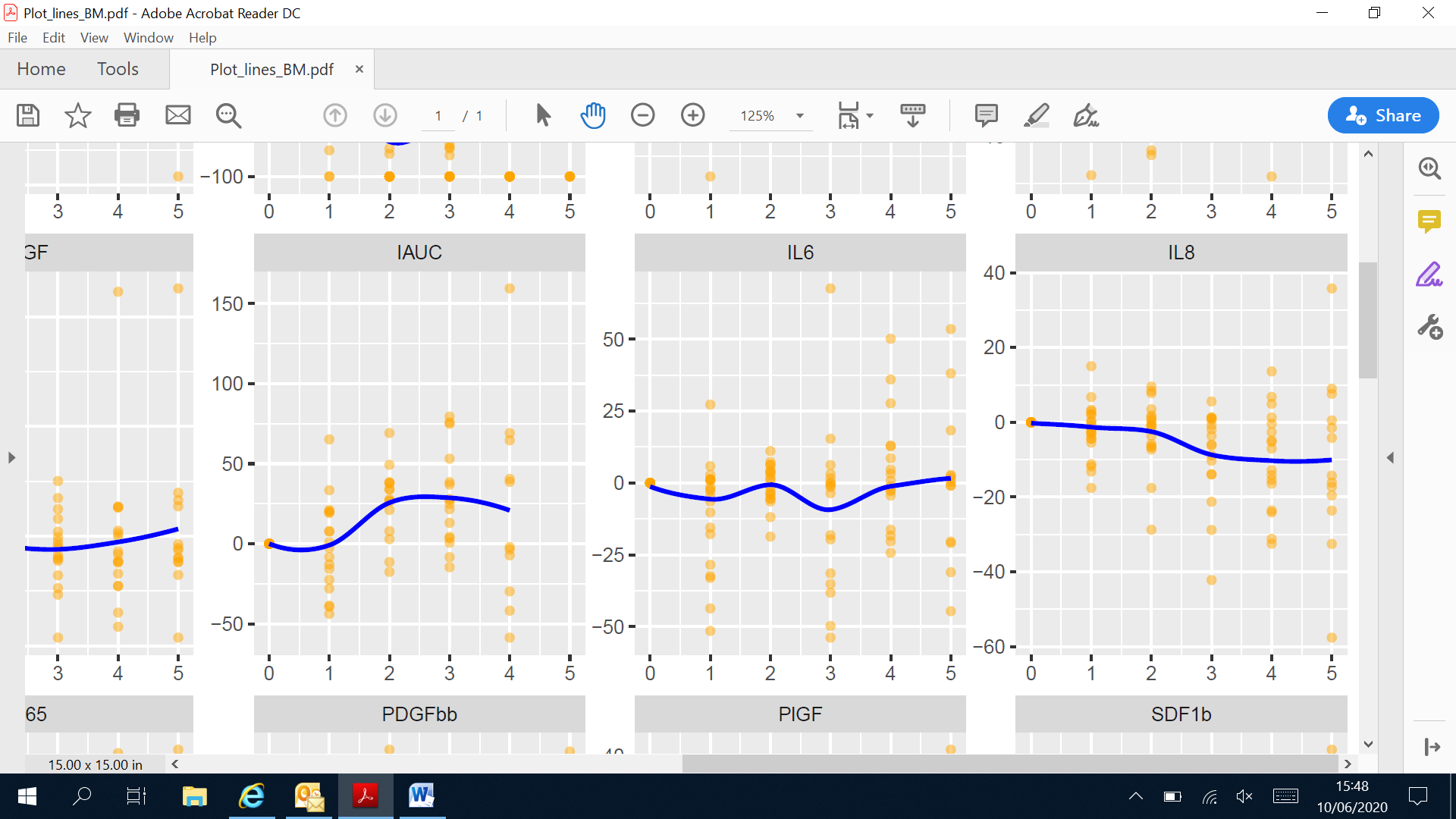


EF

ETV

iAUC

ADC


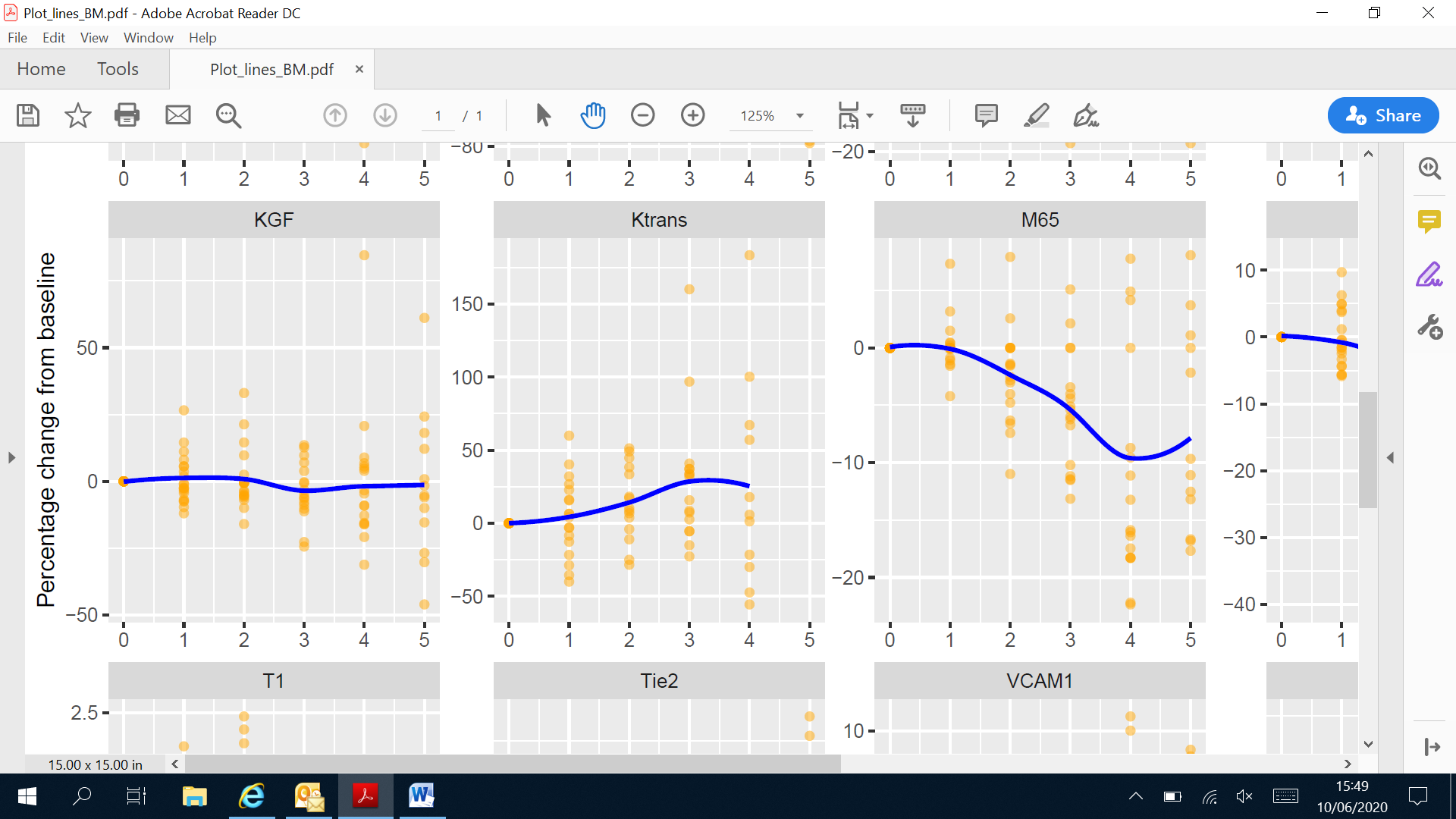

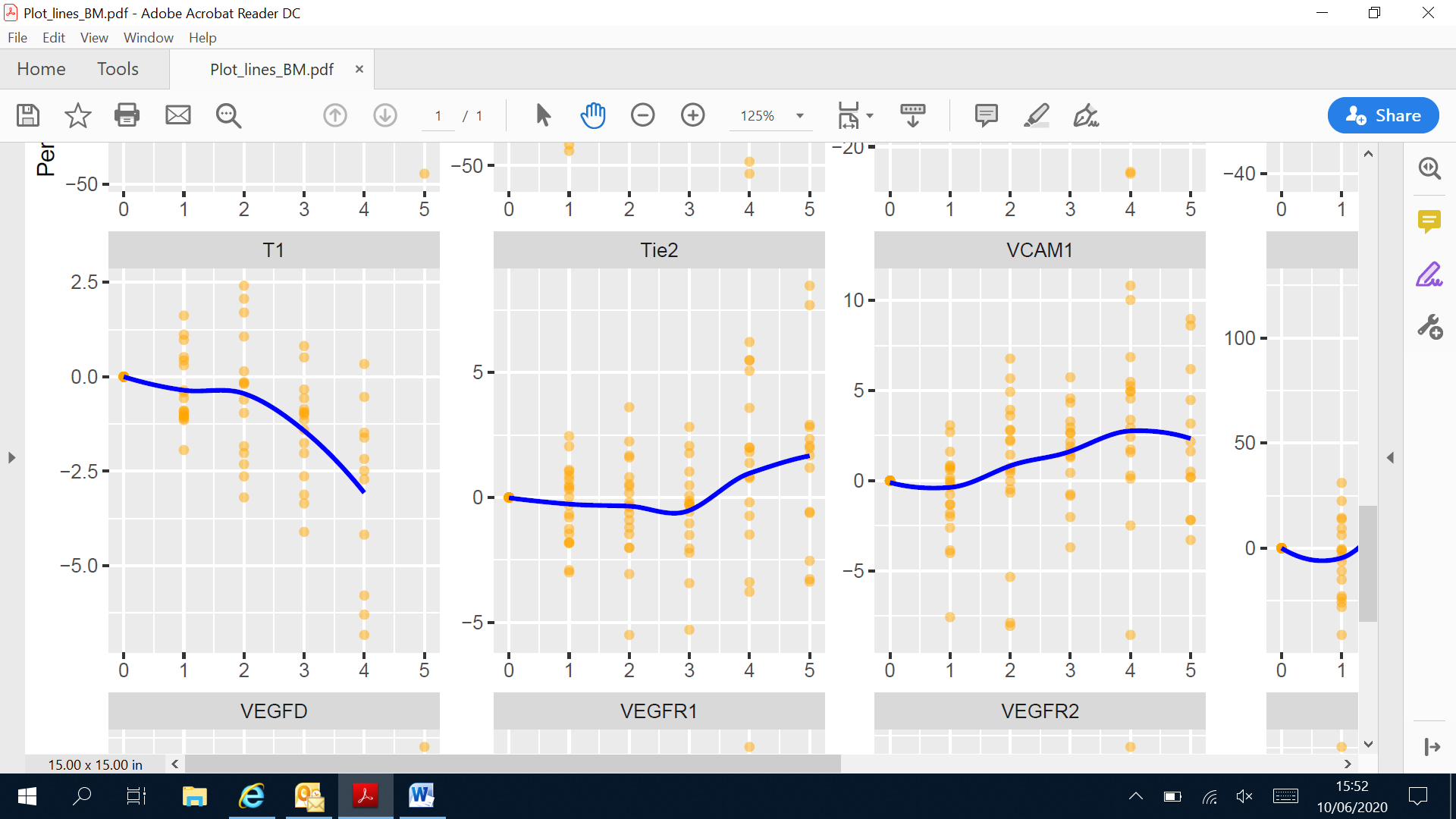

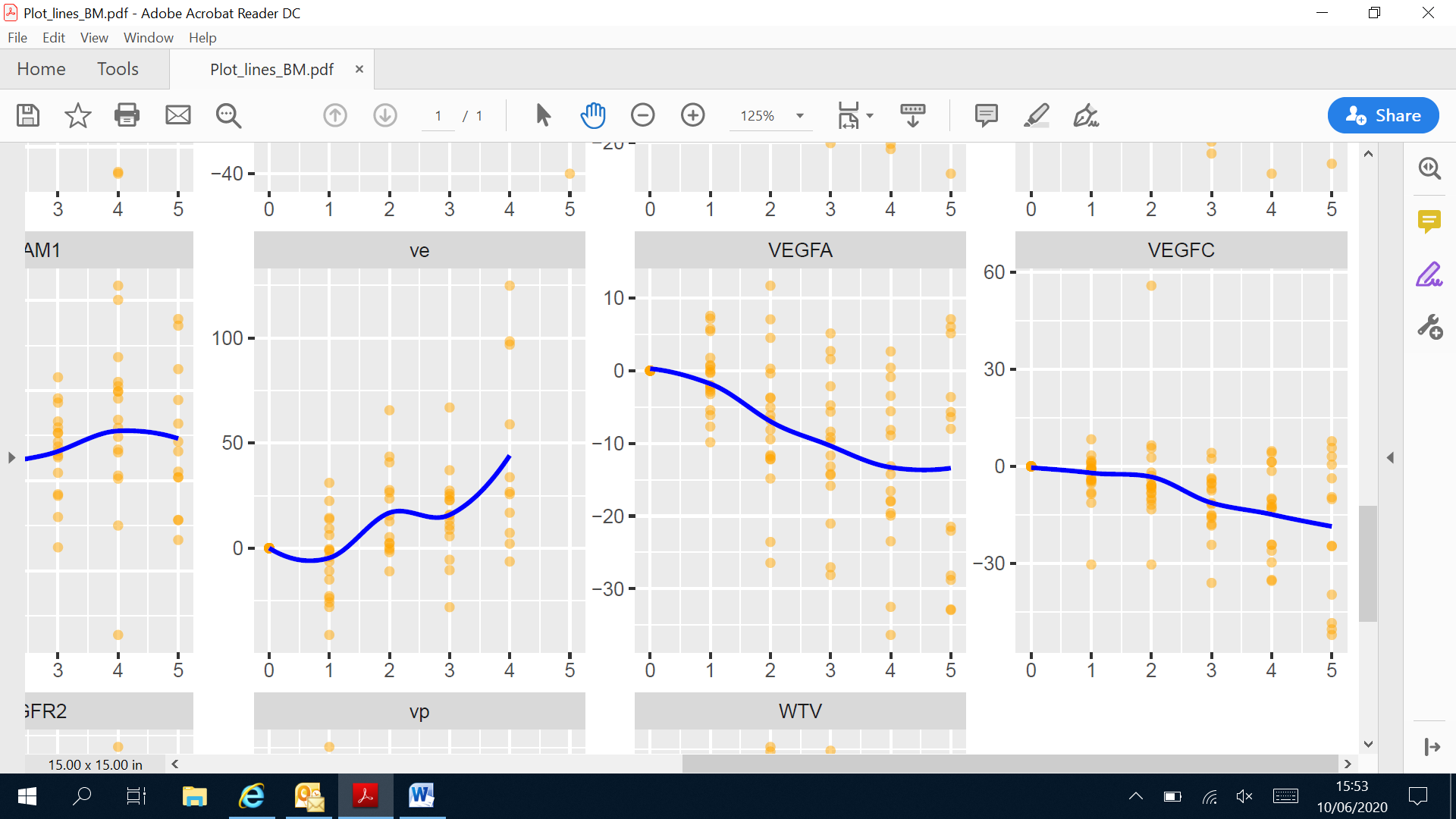

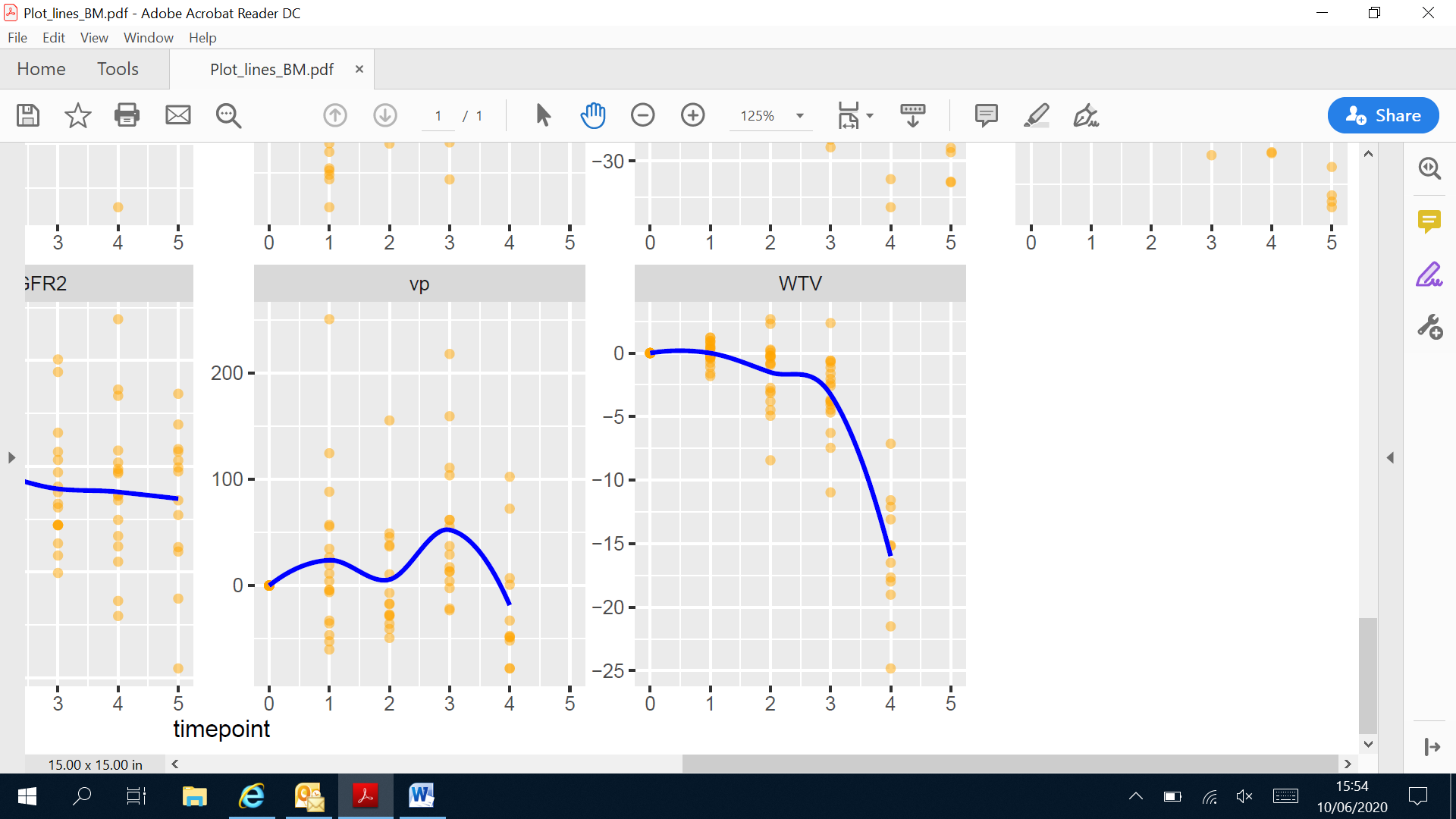


*K*^trans^

T1

ν_e_

ν_p_


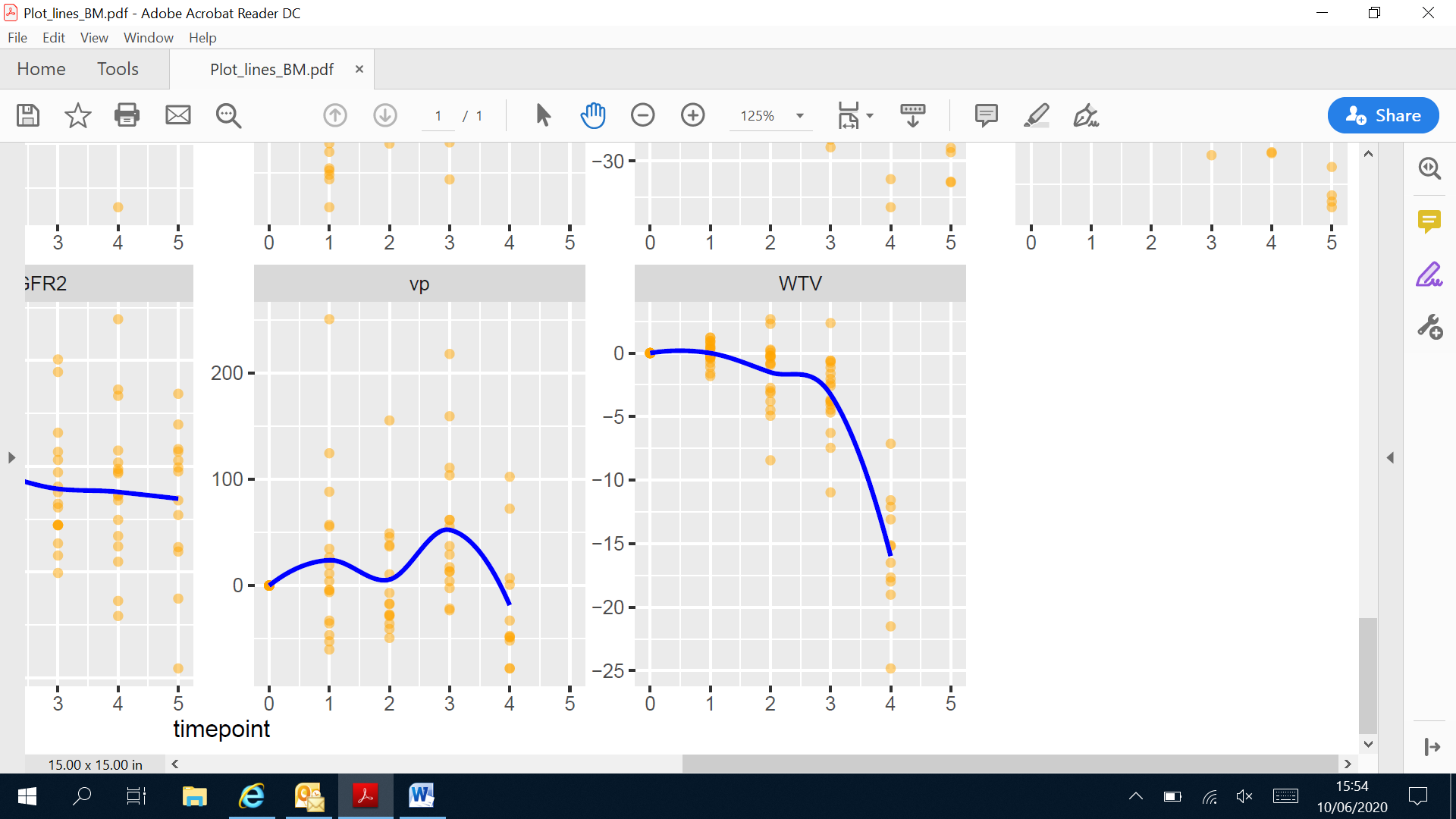


WTV

***Table 6: Change in biomarker measurement from pre-treatment to cycle 2 day 2***

Paired student t-tests are used to test whether biomarker concentration changes significantly from pre-treatment to cycle 2 day 2.

*Biomarker measurements were all log2 transformed, except ADC and *K*^trans^ which were multiplied by 100, and iAUC, ν_e_, ν_p_ and EF which were not changed.

All circulating biomarkers measured in pg/ml. Imaging biomarkers are stated. ν_e_, ν_p_ and EF have no units.

| **Biomarker type** | **Biomarker name** | **Mean change from pre-treatment to cycle 2 day 2** | **Range** | **Transformed* mean change from pre-treatment to cycle 2 day 2 [95% CI]** | **p-value** |
| --- | --- | --- | --- | --- | --- |
| Circulating | VEGF-C | -198.14 | -674.63 - 185.98 | -0.93 [-1.33 - -0.53] | 0.0002 |
|  | FGFb | -197.95 | -1362.77 - 12.79 | -0.87 [-1.26 - -0.47] | 0.0003 |
|  | VEGF-A | -88.21 | -297.98 - 31.83 | -0.79 [-1.16 - -0.42] | 0.0004 |
|  | M65 | -461.48 | -1192.13 - 234.5 | -0.57 [-0.85 - -0.3] | 0.0004 |
|  | Ang2 | -344.86 | -1329.48 - 77.12 | -0.72 [-1.08 - -0.36] | 0.0006 |
|  | Ang1 | -1107.22 | -2792.52 - 809.22 | -0.72 [-1.1 - -0.35] | 0.0009 |
|  | VEGF-D | -252.35 | -2771.22 - 397.95 | -0.32 [-0.49 - -0.14] | 0.0014 |
|  | CTCs | -4.22 | -15 - 4.5 | -1.31 [-2.07 - -0.55] | 0.0021 |
|  | PDGFbb | -144.32 | -1422.86 - 41.14 | -0.44 [-0.74 - -0.14] | 0.0070 |
|  | VCAM-1 | 386935.66 | -440847.78 - 3305175.76 | 0.32 [0.07 - 0.58] | 0.0149 |
|  | E-selectin | -2109.99 | -10941.05 - 14314.33 | -0.3 [-0.54 - -0.06] | 0.0183 |
|  | IL6 | -11.79 | -88.03 - 16.36 | -0.48 [-1.03 - 0.06] | 0.0772 |
|  | KGF | -2.43 | -14.12 - 6.12 | -0.17 [-0.37 - 0.04] | 0.1042 |
|  | VEGFR2 | -778.17 | -3430.4 - 2083.68 | -0.12 [-0.26 - 0.03] | 0.1158 |
|  | IL8 | -13.71 | -121.83 - 12.46 | -0.47 [-0.82 - -0.12] | 0.0124 |
|  | HGF | -46.7 | -424.49 - 239.02 | -0.23 [-0.52 - 0.09] | 0.1546 |
|  | VEGFR1 | 7.88 | -57.99 - 75.48 | 0.11 [-0.09 - 0.32] | 0.2640 |
|  | Tie2 | -1355.81 | -17491.13 - 6682.79 | -0.08 [-0.23 - 0.08] | 0.2963 |
|  | PlGF | -5.37 | -37.97 - 8.43 | -0.06 [-0.26 - 0.15] | 0.5657 |
|  | SDF1b | -2.04 | -1444 - 3555.69 | -0.03 [-0.27 - 0.21] | 0.8002 |
| Imaging | WTV (mm^3^) | -40774.46 | -207548.55 - 76817.69 | -0.52 [-0.75 - -0.3] | 0.0002 |
|  | ETV (mm^3^) | -38512.94 | -213454.96 - 76577.31 | -0.56 [-0.81 - -0.31] | 0.0003 |
|  | T1 (ms) | -99.53 | -325.1 - 67.73 | -0.14 [-0.22 - -0.07] | 0.0008 |
|  | iAUC (mmol.s) | 3.59 | -3.65 - 13.03 | 3.59 [1.36 - 5.81] | 0.0037 |
|  | ADC  (x10^-3^mm2/s) | 0.00 | 0.00 - 0.00 | 0.01 [0.002, 0.018] | 0.0170 |
|  | ν_e_ | 0.04 | -0.11 - 0.14 | 0.04 [0.01 - 0.07] | 0.0254 |
|  | ν_p_ | 0.01 | -0.004 - 0.04 | 0.01 [0 - 0.01] | 0.0361 |
|  | *K*^trans^(/min) | 0.03 | -0.06 - 0.22 | 3.39 [0.05 - 6.7] | 0.0469 |
|  | EF | 0.09 | -4.94 - 3.36 | 0.09 [-0.87 - 1.04] | 0.8471 |

***Table 7: Cycle 2 Day 2 biomarkers associated with progression free survival***

No biomarkers are associated with progression free survival at cycle 2 day 2, using the p-value of p<0.025 as the cut-off for significance.

Biomarker measurements were all log2 transformed, except ADC and *K*^trans^ which were multiplied by 100, and iAUC, v_e_, v_p_ and EF which were not changed.

All circulating biomarkers measured in pg/ml. Imaging biomarkers are stated. ν_e_, ν_p_ and EF have no units.

| **Biomarker type** | **Biomarker** | **HR [95% CI]** | **p-value** |
| --- | --- | --- | --- |
| Circulating | Ang2 | 0.29 [0.09 - 0.93] | 0.037 |
|  | KGF | 0.53 [0.27 - 1.03] | 0.060 |
|  | FGFb | 0.7 [0.48 - 1.04] | 0.077 |
|  | VEGF-C | 0.73 [0.5 - 1.05] | 0.088 |
|  | VEGF-A | 0.62 [0.33 - 1.14] | 0.123 |
|  | E-selectin | 0.68 [0.38 - 1.23] | 0.204 |
|  | VEGFR2 | 0.52 [0.18 - 1.45] | 0.210 |
|  | HGF | 0.54 [0.19 - 1.54] | 0.247 |
|  | VCAM-1 | 0.72 [0.34 - 1.54] | 0.398 |
|  | IL6 | 0.88 [0.66 - 1.19] | 0.411 |
|  | VEGFR1 | 0.78 [0.43 - 1.42] | 0.417 |
|  | PDGFbb | 0.74 [0.34 - 1.61] | 0.447 |
|  | CTCs | 1.26 [0.62 - 2.62] | 0.508 |
|  | PlGF | 0.91 [0.69 - 1.2] | 0.518 |
|  | VEGF-D | 0.93 [0.71 - 1.21] | 0.594 |
|  | SDF1b | 0.87 [0.5 - 1.5] | 0.616 |
|  | IL8 | 0.93 [0.66 - 1.32] | 0.684 |
|  | M65 | 1.1 [0.55 - 2.19] | 0.799 |
|  | Ang1 | 1.04 [0.61 - 1.77] | 0.886 |
|  | Tie2 | 1.01 [0.35 - 2.88] | 0.993 |
| Imaging | ν_p_ | 5.71 [8.93 - 3.66] | 0.205 |
|  | *K*^trans^ (/min) | 1.03 [0.96 - 1.1] | 0.459 |
|  | iAUC (mmol.s) | 1.03 [0.95 - 1.13] | 0.460 |
|  | ETV (mm^3^) | 1.08 [0.82 - 1.42] | 0.594 |
|  | EF | 1.08 [0.81 - 1.43] | 0.602 |
|  | ν_e_ | 3.31 [0.02 - 706.29] | 0.662 |
|  | WTV (mm^3^) | 1.05 [0.81 - 1.36] | 0.708 |
|  | T1 (ms) | 1.27 [0.2 - 8.02] | 0.803 |
|  | ADC (x10^-3^mm2/s) | 4.6 [1.05 - 2.02] | 0.924 |

***Table 8: Cycle 2 Day 2 biomarkers associated with overall survival***

At cycle 2 day 2, a higher number of CTCs is associated with worse overall survival. No other biomarkers are associated with overall survival, using the p-value of p<0.025 as the cut-off for significance.

Biomarker measurements were all log2 transformed, except ADC and *K*^trans^ which were multiplied by 100, and iAUC, v_e_, v_p_ and EF which were not changed.

All circulating biomarkers measured in pg/ml. Imaging biomarkers are stated. ν_e_, ν_p_ and EF have no units.

| **Biomarker type** | **Biomarker** | **HR [95% CI]** | **p-value** |
| --- | --- | --- | --- |
| Circulating | CTCs | 2.82 [1.3 - 6.1] | 0.008 |
|  | VEGF-C | 0.69 [0.46 - 1.02] | 0.063 |
|  | FGFb | 0.69 [0.46 - 1.04] | 0.079 |
|  | EF | 1.37 [0.95 - 1.98] | 0.094 |
|  | PDGFbb | 0.51 [0.22 - 1.18] | 0.114 |
|  | M65 | 1.7 [0.85 - 3.43] | 0.136 |
|  | E-selectin | 0.65 [0.37 - 1.15] | 0.138 |
|  | IL8 | 1.26 [0.85 - 1.86] | 0.244 |
|  | VEGF-A | 0.79 [0.47 - 1.31] | 0.359 |
|  | HGF | 0.77 [0.41 - 1.48] | 0.437 |
|  | PlGF | 1.12 [0.83 - 1.5] | 0.457 |
|  | Ang2 | 0.78 [0.39 - 1.55] | 0.472 |
|  | VEGF-D | 1.09 [0.81 - 1.48] | 0.562 |
|  | VEGFR2 | 0.79 [0.3 - 2.09] | 0.630 |
|  | Tie2 | 0.84 [0.34 - 2.07] | 0.705 |
|  | SDF1b | 1.1 [0.63 - 1.91] | 0.742 |
|  | KGF | 0.91 [0.48 - 1.74] | 0.778 |
|  | Ang1 | 0.94 [0.59 - 1.52] | 0.813 |
|  | VCAM-1 | 1.09 [0.54 - 2.17] | 0.817 |
|  | IL6 | 1.02 [0.74 - 1.42] | 0.886 |
|  | VEGFR1 | 0.99 [0.52 - 1.9] | 0.979 |
| Imaging | T1 (ms) | 2.37 [0.51 - 10.93] | 0.268 |
|  | ETV (mm^3^) | 1.13 [0.85 - 1.52] | 0.399 |
|  | iAUC (mmol.s) | 0.96 [0.88 - 1.05] | 0.409 |
|  | WTV (mm^3^) | 1.11 [0.84 - 1.45] | 0.466 |
|  | ν_e_ | 0.18 [0.001 - 51.59] | 0.555 |
|  | ν_p_ | 1.14 [3.01 - 4.29] | 0.743 |
|  | *K*^trans^ (/min) | 0.99 [0.92 - 1.06] | 0.759 |
|  | ADC (x10^-3^mm2/s) | 8.62 [1.62 - 4.6] | 0.864 |

***Table 9: Change in biomarkers associated with progression free survival***

None of the changes in biomarker concentrations from pre-treatment to cycle 2 day 2 are associated with progression free survival, using the p-value of p<0.025 as the cut-off for significance.

Biomarker measurements were all log2 transformed, except ADC and *K*^trans^ which were multiplied by 100, and iAUC, v_e_, v_p_ and EF which were not changed.

All circulating biomarkers measured in pg/ml. Imaging biomarkers are stated. ν_e_, ν_p_ and EF have no units.

| **Biomarker type** | **Biomarker** | **HR [95% CI]** | **p-value** |
| --- | --- | --- | --- |
| Circulating | PDGFbb | 3.22 [0.98 - 10.60] | 0.054 |
|  | VEGF-D | 0.32 [0.07 - 1.49] | 0.145 |
|  | IL6 | 0.66 [0.38 - 1.17] | 0.153 |
|  | Ang1 | 1.63 [0.81 - 3.27] | 0.168 |
|  | SDF1b | 0.39 [0.09 - 1.72] | 0.215 |
|  | VEGFR2 | 0.31 [0.04 - 2.18] | 0.240 |
|  | Ang2 | 1.53 [0.67 - 3.53] | 0.316 |
|  | Tie2 | 2.43 [0.35 - 16.9] | 0.369 |
|  | VEGF-C | 0.68 [0.26 - 1.77] | 0.432 |
|  | M65 | 1.41 [0.57 - 3.51] | 0.461 |
|  | HGF | 1.38 [0.54 - 3.55] | 0.500 |
|  | KGF | 0.61 [0.12 - 3.22] | 0.564 |
|  | VCAM-1 | 0.79 [0.29 - 2.16] | 0.642 |
|  | IL8 | 1.21 [0.53 - 2.76] | 0.649 |
|  | FGFb | 0.86 [0.4 - 1.86] | 0.706 |
|  | VEGFR1 | 1.26 [0.36 - 4.46] | 0.722 |
|  | PlGF | 1.2 [0.31 - 4.7] | 0.796 |
|  | VEGF-A | 1.06 [0.45 - 2.47] | 0.897 |
|  | CTCs | 0.98 [0.67 - 1.43] | 0.929 |
|  | E-selectin | 0.96 [0.34 - 2.69] | 0.941 |
| Imaging | ETV (mm^3^) | 5.2 [0.84 - 32.12] | 0.076 |
|  | T1 (ms) | 0.02 [0 - 2.77] | 0.118 |
|  | ν_e_ | 0 [0 - 9.55] | 0.125 |
|  | WTV (mm^3^) | 2.84 [0.67 - 12.03] | 0.156 |
|  | ν_p_ | 1.67 [1.08 - 2.6] | 0.521 |
|  | ADC (x10^-3^mm2/s) | 4.78 [1.1 - 2.07] | 0.531 |
|  | EF | 1.08 [0.81 - 1.43] | 0.603 |
|  | *K*^trans^ (/min) | 1.03 [0.92 - 1.15] | 0.658 |
|  | iAUC (mmol.s) | 1.01 [0.85 - 1.19] | 0.944 |

***Table 10: Change in biomarkers associated with overall survival***

An increase in *K*^trans^ from pre-treatment to cycle 2 day 2 is significantly associated with worse overall survival outcome, but not other biomarkers are associated with overall survival using the p-value of p<0.025 as the cut-off for significance.

Biomarker measurements were all log2 transformed, except ADC and *K*^trans^ which were multiplied by 100, and iAUC, v_e_, v_p_ and EF which were not changed.

All circulating biomarkers measured in pg/ml. Imaging biomarkers are stated. ν_e_, ν_p_ and EF have no units.

| **Biomarker type** | **Biomarker** | **HR [95% CI]** | **p-value** |
| --- | --- | --- | --- |
| Circulating | E-selectin | 0.26 [0.07 - 0.89] | 0.032 |
|  | IL6 | 0.58 [0.34 - 0.98] | 0.040 |
|  | VEGF-C | 0.5 [0.23 - 1.05] | 0.067 |
|  | VCAM-1 | 0.52 [0.2 - 1.39] | 0.196 |
|  | M65 | 0.68 [0.29 - 1.63] | 0.388 |
|  | Tie2 | 2.05 [0.33 - 12.61] | 0.438 |
|  | PlGF | 0.64 [0.21 - 1.99] | 0.440 |
|  | KGF | 0.66 [0.22 - 1.95] | 0.451 |
|  | FGFb | 0.87 [0.52 - 1.44] | 0.582 |
|  | VEGFR2 | 1.48 [0.24 - 9.29] | 0.678 |
|  | VEGF-A | 0.91 [0.51 - 1.63] | 0.748 |
|  | HGF | 0.93 [0.49 - 1.74] | 0.812 |
|  | CTCs | 1.04 [0.68 - 1.61] | 0.848 |
|  | PDGFbb | 0.96 [0.39 - 2.37] | 0.934 |
|  | Ang2 | 1.02 [0.57 - 1.81] | 0.953 |
|  | VEGF-D | 1.04 [0.23 - 4.67] | 0.955 |
|  | Ang1 | 0.99 [0.55 - 1.78] | 0.964 |
|  | VEGFR1 | 0.97 [0.22 - 4.2] | 0.967 |
|  | IL8 | 0.99 [0.47 - 2.08] | 0.979 |
|  | SDF1b | 0.99 [0.3 - 3.32] | 0.993 |
| Imaging | *K*^trans^ (/min) | 1.1 [1.01 - 1.2] | 0.025 |
|  | EF | 1.49 [1.05 - 2.11] | 0.026 |
|  | iAUC (mmol.s) | 1.18 [1.01 - 1.36] | 0.034 |
|  | ETV (mm^3^) | 3.4 [0.46 - 25.08] | 0.230 |
|  | T1 (ms) | 0.15 [0.003 - 6.5] | 0.320 |
|  | WTV (mm^3^) | 1.97 [0.44 - 8.95] | 0.379 |
|  | ν_e_ | 17.62 [0.002 - 127251.76] | 0.527 |
|  | ν_p_ | 5.62 [1.12 - 2.83] | 0.592 |
|  | ADC (x10^-3^mm2/s) | 5.8 [2.001 - 1.68] | 0.638 |

1. **REFERENCES**

1. UK Legislation. The Medicines for Human Use (Clinical Trials) Regulations 2004 [Internet]. 2004 [cited 2020 May 12]. Available from: http://www.legislation.gov.uk/uksi/2004/1031/pdfs/uksi_20041031_en.pdf

2. O’Connor JPB, Carano RAD, Clamp AR, Ross J, Ho CCK, Jackson A, et al. Quantifying Antivascular Effects of Monoclonal Antibodies to Vascular Endothelial Growth Factor: Insights from Imaging. Clin Cancer Res [Internet]. 2009 Nov 1;15(21):6674–82. Available from: http://clincancerres.aacrjournals.org/cgi/doi/10.1158/1078-0432.CCR-09-0731

3. Parker G, Jackson A, Waterton J, Buckley D. Automated Arterial Input Function Extraction for T1-Weighted DCE-MRI. In: In Proceedings of the ISMRM eleventh meeting Toronto. 2003.

4. Tofts PS. Modeling tracer kinetics in dynamic Gd-DTPA MR imaging. J Magn Reson Imaging [Internet]. 1997 Jan;7(1):91–101. Available from: http://doi.wiley.com/10.1002/jmri.1880070113

5. Kagan M, Howard D, Bendele T, Mayes J, Silvia J, Repollet M, Doyle J, Allard J, Tu N, Bui T, Russell T, Rao C, Hermann M RH and TL. A sample preparation and analysis system for identification of circulating tumor cells. J Clin Lig Assay. 2002;25:104–10.

6. Backen A, Renehan AG, Clamp AR, Berzuini C, Zhou C, Oza A, et al. The Combination of Circulating Ang1 and Tie2 Levels Predicts Progression-Free Survival Advantage in Bevacizumab-Treated Patients with Ovarian Cancer. Clin Cancer Res [Internet]. 2014 Sep 1;20(17):4549–58. Available from: http://clincancerres.aacrjournals.org/cgi/doi/10.1158/1078-0432.CCR-13-3248

7. Greystoke A, Dean E, Saunders MP, Cummings J, Hughes A, Ranson M DC and RA. Multi-level evidence that circulating CK18 is a biomarker of tumour burden in colorectal cancertle. Br J Cancer. 2012;107(9):1518–24.

8. Cummings J, Ranson M, Lacasse E, Ganganagari JR, St-Jean M, Jayson G DJ and DC. Method validation and preliminary qualification of pharmacodynamic biomarkers employed to evaluate the clinical efficacy of an antisense compound (AEG35156) targeted to the X-linked inhibitor of apoptosis protein XIAP. Br J Cancer. 2006;95(1):42–8.
